# Unleashing the potential: AI empowered advanced metasurface research

**DOI:** 10.1515/nanoph-2023-0759

**Published:** 2024-02-27

**Authors:** Yunlai Fu, Xuxi Zhou, Yiwan Yu, Jiawang Chen, Shuming Wang, Shining Zhu, Zhenlin Wang

**Affiliations:** National Laboratory of Solid State Microstructures, School of Physics, School of Electronic Science and Engineering, Nanjing University, Nanjing 210093, China; National Laboratory of Solid State Microstructures, School of Physics, Nanjing University, Nanjing 210093, China; Collaborative Innovation Center of Advanced Microstructures, Nanjing 210093, China

**Keywords:** metasurface, machine learning, inverse design, computational imaging

## Abstract

In recent years, metasurface, as a representative of micro- and nano-optics, have demonstrated a powerful ability to manipulate light, which can modulate a variety of physical parameters, such as wavelength, phase, and amplitude, to achieve various functions and substantially improve the performance of conventional optical components and systems. Artificial Intelligence (AI) is an emerging strong and effective computational tool that has been rapidly integrated into the study of physical sciences over the decades and has played an important role in the study of metasurface. This review starts with a brief introduction to the basics and then describes cases where AI and metasurface research have converged: from AI-assisted design of metasurface elements up to advanced optical systems based on metasurface. We demonstrate the advanced computational power of AI, as well as its ability to extract and analyze a wide range of optical information, and analyze the limitations of the available research resources. Finally conclude by presenting the challenges posed by the convergence of disciplines.

## 1 Introduction

Optics is an ancient discipline, and it is the most important means used by humans to obtain information about the real world. The function of traditional optical elements is based on the effects of reflection [1], [2], [3], refraction [4], [5], diffraction [6], [7], [8], dispersion [9], [10], and their combinations. Due to the limitations of natural materials for light modulation, multiple elements are usually required to be combined so as to satisfy the desired function, making it difficult to lighten traditional optical systems. Optics has a broad application prospect in the fields of communication [11], [12], [13], imaging [14], [15], [16], display [17], [18], [19], etc. but is limited by the complexity of computation and the difficulty of integrating various types of components. To this end, the metasurface [20], [21], [22]was emerged, which is a planar optical element that can control light on the sub-wavelength scale and directly manipulate electromagnetic waves. Metasurface consists of a certain arrangement of meta-atom arrays, which can flexibly regulate the amplitude [23], [24], phase [25], [26], [27], polarization [28], [29], and other physical parameters [30], [31]. From material selection [32], [33], atom shape design [34], [35], adjusting phase distribution [36], designing array arrangement [37], [38], [39], and back-end processing [40], [41], [42], etc., the functions of metasurface can be independently regulated, which makes metasurface become an important development direction for future advanced flat optics [43].

The design of metasurfaces exhibits high degrees of freedom (DoF) and diverse functionalities, but there still exists a certain gap between theoretical design and practical applications, with limited computational capabilities and resources being one of the important limiting factors. On one hand, the fundamental theory of metasurfaces is not yet well-established. Although the wavefront manipulation of metasurfaces can be described by the generalized Snell’s Law [22], the phase of each meta-atom at different positions has unique physical forms. For example, metal-based metasurfaces rely on surface plasmon resonances [44], [45], resonant excitations [46], and electromagnetic field enhancement [47], [48], while dielectric metasurfaces are based on resonances of electric and magnetic dipoles [49], [50]. Additionally, there is the Pancharatnam–Berry (PB) phase [51], [52] that is independent of the material and enables polarization conversion of circularly polarized incident light. The electromagnetic response of meta-atoms is influenced by various factors, resulting in various scattering effects and exciting new effects under linear or nonlinear states, making it difficult to describe the changes in the electromagnetic field in space concisely and objectively. Directly solving Maxwell’s equations is the most basic way to solve the electromagnetic distribution problem of metasurface, but it is challenging for humans, even with the development of simplified algorithms such as finite difference time-domain method (FDTD) [53], [54], [55] and rigorous coupled-wave analysis (RCWA) [56], [57], [58], which still require significant computational effort. On the other hand, although metasurface can overcome the bulkiness of traditional optical elements, their actual optical performance often falls short of the capabilities of existing complex systems in many cases. Moreover, the fabrication and characterization processes of metasurface also limit their performance. Overcoming the effects of manufacturing errors and applying theoretically designed metasurface to various nonideal application conditions are pressing challenges.

In recent years, Artificial Intelligence (AI) has experienced a rise in interdisciplinary integration, and the fusion of AI and metasurface is a highly advantageous research direction. The emergence of AI can be traced back to the 1950s [59], [60], and its ultimate goal is to enable machines to imitate human-like thinking processes. AI has been widely applied in various aspects of our lives, from computer vision [61], [62], speech recognition [63], [64], and financial analysis [65], [66] to autonomous driving [67], intelligent disease diagnosis [68], [69], [70], [71], and conversational models [72], [73]. By applying AI to specific tasks, it reduces the workload on humans and unleashes greater productivity.

An obvious fact is that AI is better suited to handle problems that are difficult for humans but straightforward for computers, such as those described by a large number of mathematical or physical rules. From a hardware perspective, the storage capacity and processor performance continue to improve, especially with the advent of GPUs [74], [75], which provide the foundation for AI to learn from big data. On the software side, humans have proposed various AI learning algorithms for different tasks, further enhancing the ability of AI to process information. The fusion of AI and metasurface introduces a new research paradigm: traditional numerical calculations can provide abundant data for AI, and AI can learn more features from these data. The two complement each other, greatly accelerating the development of metasurface. AI not only enriches the methods for metasurface element design and provides efficient optimization design approaches but also enhances the functionalities of various systems based on metasurface. It has the potential to surpass most traditional optical systems and inject new vitality into the development of the next generation of micro–nano optics.

In this article, we start with a review of the concepts of metasurface and explore the foundations for integrating AI and metasurface research. In Section 2, we briefly introduce metasurface, including traditional design methods and common applications. Then in order to make researchers unfamiliar with AI have a quick knowledge, we focus on AI and talk about some of the common conceptual terms and the underlying math of AI. In Section 3 and 4, we show how AI facilitates metasurface research. Section 3 summarizes a variety of metasurface designed by machine learning algorithms. Section 4 is dedicated to displaying AI-enhanced metasurface systems from imaging to innovative functions, which show AI’s powerful ability to extract physics information. Finally, we make a conclusion and outlook by exploring the limits and challenges of AI for metasurface in Section 5.

## 2 Basic concepts

### 2.1 Metasurface

The advantage of metasurfaces over traditional optical devices lies in their ability to control electromagnetic waves’ phase through subwavelength-thick nanoantennas on the surface of the medium. Phase modulation enables the independent control of amplitude, phase, and polarization state. The primary forward modulation method for metasurfaces is phase modulation, which includes three phase design approaches: resonant phase, propagating phase, and geometric phase (PB-phase).

**Figure 1:**
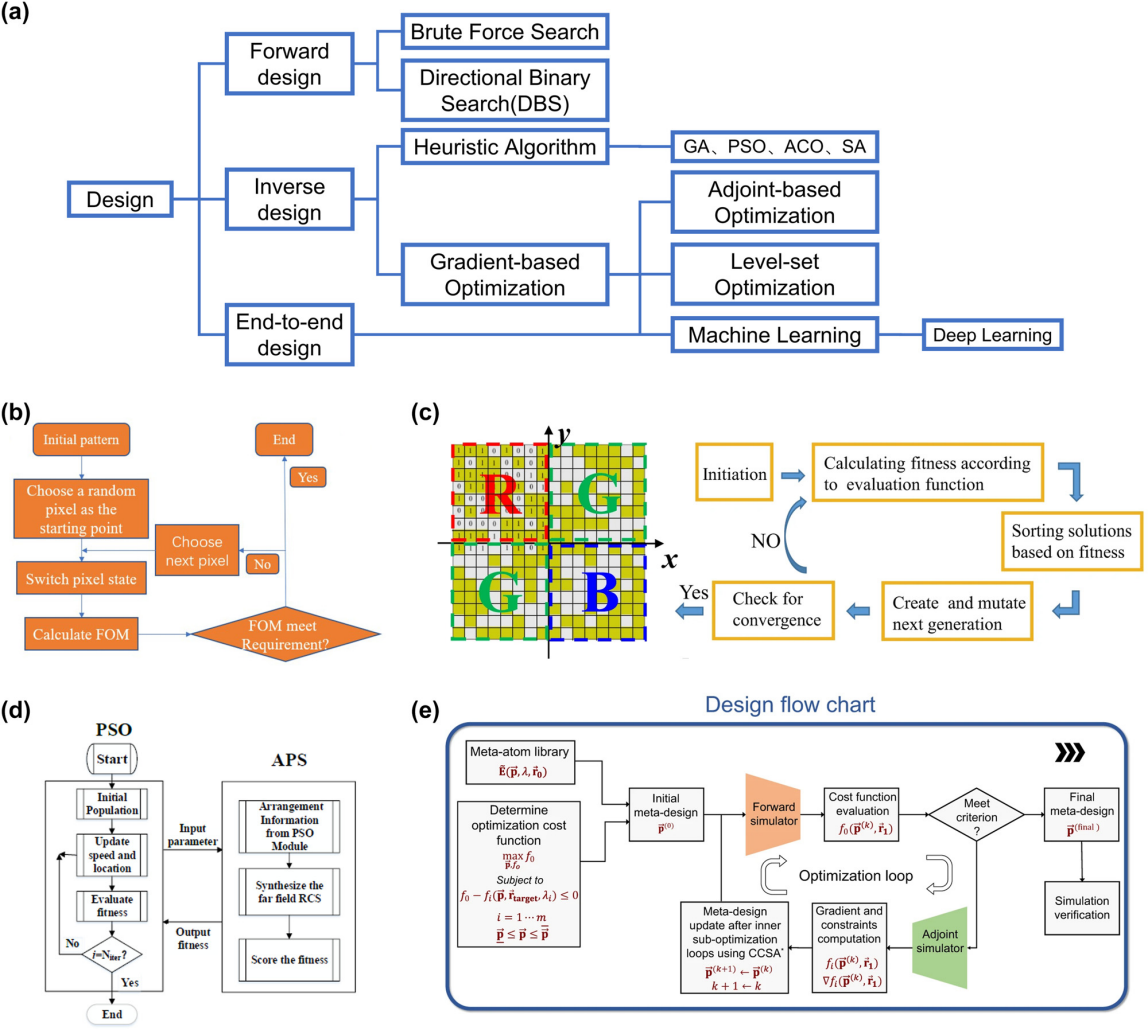
Different design methods of metasurface. (a) Different types of metasurface design algorithms. (b) Flowchart of direct binary search (DBS) algorithm for designing metasurface-based power splitter [78]. (c) Flowchart of designing a Bayer-type metasurface color router by genetic algorithm. The Quick Response Code (QR Code)-like structure was abstracted into a binary string and updated by GA [80]. (d) Flowchart of particle swarm optimization algorithm combined with Array Pattern Synthesis (APS). PSO evacuated the fitness and then updated particle velocity and population location. APS calculated radar cross section as fitness and returned to PSO module [86]. Reproduced courtesy of The Electromagnetics Academy. (e) Flowchart of adjoint-based optimization. It can evacuate the gradient of all parameters using forward and backward simulation [91].

The general design of metasurfaces can be categorized into forward design, inverse design, and end-to-end design. As shown in Figure 1(a), forward design involves modifying the shape, material, and geometric parameters of subwavelength structures and then matching the resulting characteristics with appropriate applications to meet the phase control requirements for different applications. Brute force search [76], [77] performs a parametric search to obtain the optical response of individual meta-atom, identifying valid meta-atoms to design the scale structure through the design space provided by a large number of parameters as the number of functions increases. And direct binary search (DBS) [78] is a direct design method based on numerical iterations. It first constructs the optimization objective (figure of merit, FoM) for evaluation and randomly generates the initial structure. During the execution of the algorithm, the FoM is computed by continuously inverting the pixels and calculating the FoM until the iteration limit is reached, as shown in Figure 1(b). However, the forward design approach often requires obtaining the electromagnetic response of unit structures based on boundary conditions and arranging the unit structures according to certain rules to complete the metasurface design. This method is time-consuming, difficult to optimize performance, and may fail to achieve the desired results, such as the requirements for beam control in nonlinear optics. Inverse design, on the other hand, is target-driven and involves algorithmic optimization of feasible solutions. It has become an important method for designing metasurface structures. The method requires determining the desired quantitative characteristics for system control objectives, selecting appropriate optimization algorithms, and finally simulating the optimal metasurface structure. Various optimization algorithms have been used for the modification and optimization of metasurface structures [79], which can be departed as two categories: heuristic algorithms (HAs) like genetic algorithm (GA) [80], [81], [82], [83], [84], particle swarm optimization (PSO) [85], [86], [87], ant colony optimization (ACO) [88], simulated annealing (SA) [89], [90], etc., and gradient-based algorithms like adjoint-based algorithms [91], [92], [93], [94] and level-set optimization [95], [96], [97]. The design of metasurface is a complex optimization problem with a very large search space, and HAs can search in a large-scale solution space to find approximately optimal solutions. For some problems without clear expressions or gradient information, evolutionary algorithms have the ability to find multiple local optimal solutions and may find potential optimal solutions. As a representative, GA mimics DNA variations and iterates through operations such as crossover and mutation to evolve the population toward better solutions, thereby approaching the optimal solution. Figure 1(c) shows an optimized metasurface structure using a genetic algorithm. GA is suitable for discrete parameterized designs or where complex constraints need to be considered to find the optimal solution through genetic manipulation and selection mechanism. As shown in Figure 1(d), PSO is based on intelligent optimization of a population, simulating the foraging behaviors of birds to find the optimal solution from feasible alternatives. PSO uses a certain number of particles to search for the global optimal solution. Its principle is relatively simple, and easy to implement, but tends to converge to local optima, with slower convergence speed and lower accuracy compared to GA. PSO is mainly used to solve continuous optimization problems, that is, to search for optimal solutions in real number space. And its search ability in high-dimensional optimization problems is relatively strong. HAs require a large number of populations for each iteration, resulting in significant computational complexity. In contrast, the adjoint-based methods only require one forward simulation and one adjoint simulation to obtain gradient information for the entire parameter optimization space. The adjoint-based methods can be divided into topology optimization and boundary optimization [98]. Topology optimization usually focuses on the distribution of dielectric constants, where the initial solution needs to be continuous, and then, through hundreds of iterations, the continuous distribution evolves into a binarized distribution with practical significance. Boundary optimization directly optimizes the boundaries of the device structure, allowing for further efficiency improvement with a small number of iterations based on an already binarized initial structure, as shown in Figure 1(e). The adjoint-based optimization method is more suitable for free-form metasurface optimization designs: free from hand-engineered designs schemes or free-from geometric shapes in the real space [99]. The main advantage of adjoint-based optimization is that it can quickly converge to a local minimum, which has significantly improved the optimization of structural shapes with extremely many parameters. Machine learning is essentially a gradient-based algorithm, but it leads to new design methods. Unlike forward or inverse design, end-to-end design is a means of optimizing structure and response simultaneously [100]. As AI algorithms receive more and more attention in the design of metasurface, end-to-end design has become a new trend.

Compared to traditional techniques, the control methods, flexible electromagnetic manipulation capabilities, as well as the characteristics of ultrathin, lightweight, and multifunctional integration make metasurface hold tremendous potential and prospects in the field of optics [101]. In recent years, nano-optical materials have been widely applied in optical imaging [26], [102], [103], [104], [105], holographic projection [106], [107], [108], filters [109], [110], [111], absorbers [112], [113], and other fields.

**Figure 2:**
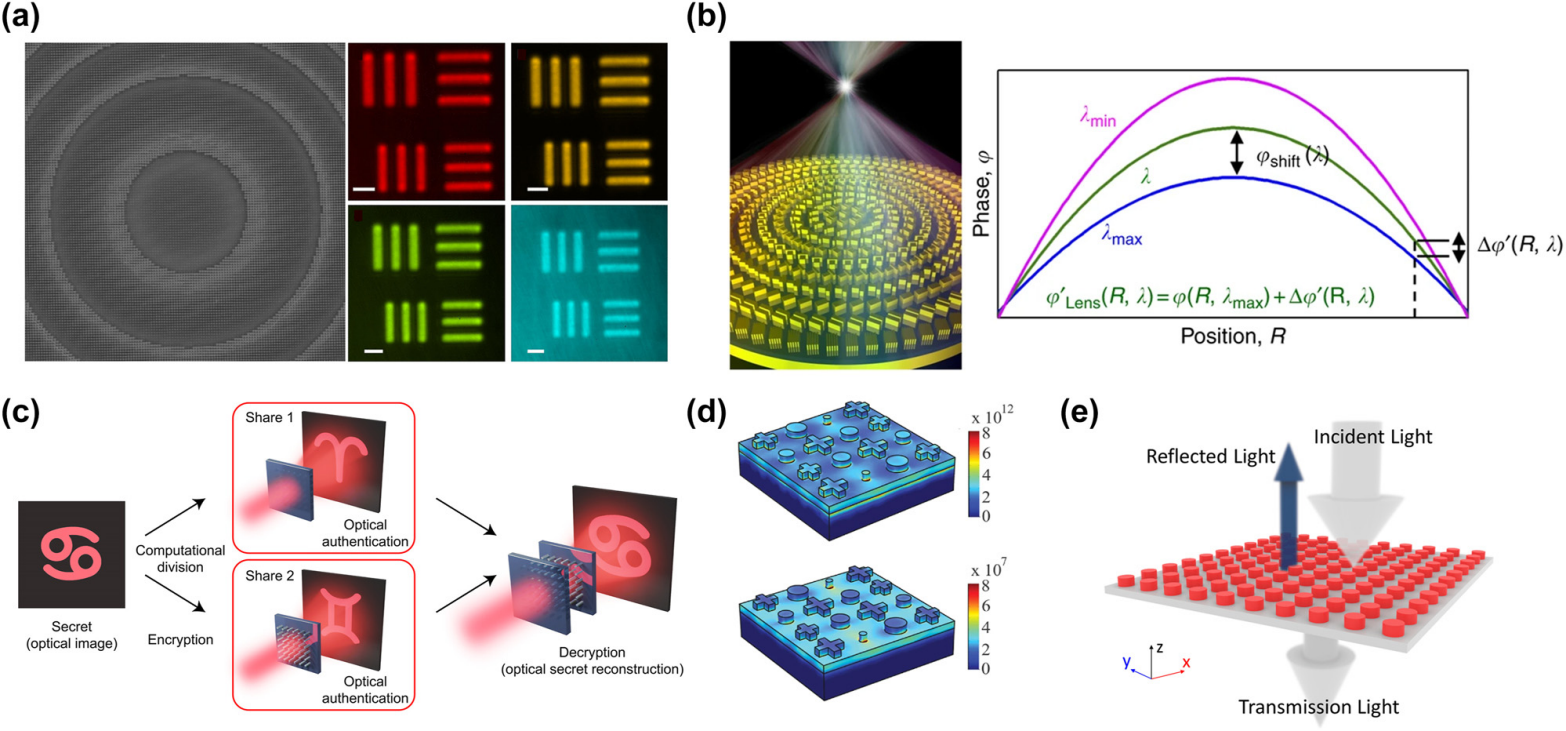
Classical metasurface applications. (a) Polarization-insensitive metalens at visible wavelength, including top-view scanning electron microscope (SEM) image of fabricated metalens and imaging with the metalens. Reproduced from [103]. Copyright 2016, American Chemical Society. (b) A broadband achromatic optical metalens and phase profile methods [114]. (c) The conceptual description of holographic secret sharing through metasurface [107]. (d) Simulated moduli of current and electric field on the surface of metasurface absorber for impinging wavelength of 350 nm [111]. (e) Schematic of the refractive metasurface as a filter [113].

Planar optics components composed of subwavelength structured metasurfaces can achieve optical focusing, achieving similar effects as traditional lenses. Khorasaninejad et al. [103] fabricated metasurface structures composed of closely arranged nanocolumns using rectangular TiO_2_ in 2016, and metalens made from such planar structures exhibited excellent optical properties, as shown in Figure 2(a). Subsequently, they demonstrated a polarization-insensitive metalens in the visible light range with a thickness of less than 600 nm, capable of focusing incident light to diffraction-limited spots as small as 0.64 and providing high-resolution imaging [26]. With the development of metalens applications, optimizing the performance of metasurface by controlling dispersion has become a key focus in the research of metalens, including the elimination of chromatic aberration and the introduction of hyperchromatism. In 2018, Wang et al. [102] demonstrated color imaging of metalens by arranging functional units based on geometric and propagating phases, achieving achromatic for both white light and colored light. As shown in Figure 2(b), the required phase for metalens focusing was split into a fundamental phase and a compensating phase, corresponding to wavelength-independent focusing and wavelength-dependent chromatic aberration, respectively. In 2021, Daniel Lim et al. [105] achieved simultaneous control of propagating phase and amplitude by inverse designing the effective refractive index of metasurfaces, improving the impedance matching between incident waves and transmitted waves, thereby enhancing the focusing efficiency. Additionally, by increasing the aspect ratio of nanostructures, the range of chromatic dispersion delay can be increased, ultimately enabling chromatic aberration correction in metalenses and further advancing the research on achromatic lenses.

Traditional holographic imaging requires a large number of optical components for phase accumulation during propagation, leading to issues of low-resolution imaging and higher-order diffraction. In 2015, Zheng et al. [106] demonstrated a geometric phase-based metasurface for reflective holographic imaging, achieving a diffraction efficiency of around 80 % at approximately 800 nm, addressing the issue of low efficiency when metasurfaces are used for hologram generation. Moreover, metasurface-based holographic imaging can also be used as spatial filters, beam shaping elements, deflectors, beam splitters, and optical interconnects. It finds applications in optical communication, high-performance computing, as well as information encryption and protection, enhancing the practicality of metasurface optical devices [108]. Philip Georgi et al. [107] designed an information-sharing encryption platform based on metasurface holographic imaging, employing complex amplitude and phase holograms to increase the complexity and security of encrypted information. The design flexibility of metasurface stems from various possible nanoscale structure shapes. Based on metasurface holographic imaging, as shown in Figure 2(c), different holographic images can be encoded along multiple optical dimensions, enhancing information security.

Subwavelength-scale metasurface absorbers possess excellent properties and have great potential in many applications, such as sensing, compressed imaging, and thermal management. Guo et al. [109] designed a broadband infrared absorber based on metasurface, achieving a total absorption efficiency exceeding 90 % in the wavelength range of 7.8–12.1 μm using a single-layer metasurface structure. They further investigated the use of a two-layer metasurface, significantly expanding the absorption bandwidth. In the same year, Azad et al. [111] designed metasurface broadband absorbers covering the entire solar spectrum based on a metal–dielectric–metal structure, and in the experimental results, the absorption efficiencies in the visible and near-infrared bands (350 nm–1100 nm) were more than 90 %, shown in Figure 2(d). Liu et al. [110] proposed a terahertz all-dielectric metasurface absorber based on hybrid dielectric waveguide resonances. This research progress provides a new approach for controlling the emission and absorption of surface electromagnetic radiation.

Traditional filters are designed based on the Fresnel formula and are composed of bulky multilayer dielectric films, which are not conducive to the application of filters in small integrated optical devices. Ghasemi [112] designed a metasurface based on concentric annular resonators, demonstrating the possibility of using metasurface structures for filter fabrication. Shen et al. [113] proposed and designed a dielectric metasurface filter composed of a silicon nano-disc array. This filter operates under the resonance excitation of electric dipoles. Due to its symmetry, the dielectric metasurface is independent in terms of polarization and exhibits high efficiency in the mid-infrared wavelength range, which is highly sensitive to the operating frequency. Figure 2(e) shows a perspective view of the filter.

At present, most of the classic applications of metasurfaces are goal-driven, and their design scope still follows the concept of traditional optical components, that is, according to the optical response requirements of specific problems, the corresponding physical parameters can be controlled by optimizing different structures. Compared with traditional optical elements, metasurfaces have a higher degree of control freedom, allowing metasurfaces to achieve higher-quality imaging, multidimensional imaging or more diverse applications, which is its biggest advantage. However, the search capability of large parameter space that comes with it limits its development. AI technology, especially deep learning, can process and identify complex patterns and features and has excellent information extraction capabilities for nonlinear optical responses. Applying AI to the design of metasurfaces, by learning from large amounts of data, the design parameters of the metasurface can be adaptively adjusted, and it can adapt to different inputs and environmental conditions. This data-driven solution is no longer limited to a single function but can tap the potential structure and performance in metasurface design, thus having great application potential in the fields of imaging, sensing, and communications.

### 2.2 Artificial intelligence

The origin of Artificial Intelligence (AI) can be traced back to 1950 when Alan Turing published a paper titled “Computing Machinery and Intelligence.” In this paper, he constructed a philosophical framework to explore the concept of “thinking machines” and proposed three different strategies to achieve this goal. Six years later, the Dartmouth Conference was held, where John McCarthy officially coined the term “Artificial Intelligence” to distinguish it from another independent field of science, namely cybernetics [115]. AI is an important field in computer science that aims to study how to mimic and replicate human-like behavior, processes, and strategies for problem-solving using software and hardware. The development of AI has gone through three major stages. The first stage, in the 1950s and 1960s, focused on developing heuristic algorithms and programs for solving creative and intellectual problems. The second stage, from the late 1960s to the early 1970s, aimed to create intelligent robots capable of modeling the external world, recognizing, evaluating, and making decisions, and eventually forming behavioral plans and engaging in natural language communication. The third stage, from the mid-1970s to the present, is characterized by the research and development of intelligent human–computer systems that integrate human intelligence with computer capabilities. In this evolutionary process, a significant milestone was reached in 2012 when Alex Krizhevsky used Convolutional Neural Networks (CNN) to outperform shallow machine learning methods in a competition, marking the arrival of the era of deep learning [116]. The rise of deep learning has brought significant breakthroughs to AI, as it can extract complex features from large amounts of data through hierarchical representation learning, achieving important advancements in image recognition, natural language processing, and other fields. The development of these stages demonstrates the continuous progress and innovation in the field of AI, bringing us wide-ranging applications and tremendous potential.

The intersection and application of AI and physics have yielded diverse achievements. Then the characteristics of AI give it a significant advantage in optimization and simulation, involving fields such as material discover and design [117], [118], drug synthesis [119], simulation of quantum many-body systems [120], physics simulation [121], equation exploration [122], etc. At the same time, AI also contributes to the utilization of physics knowledge in computing new hardware or new paradigms, such as using optical devices for neural network computing [123], optical computing aspects of artificial intelligence applications [124], as well as applications in audio and image processing [125], [126]. This interdisciplinary fusion brings enormous potential and innovation, propelling the development of AI and physics.

AI is a vast research field, and machine learning (ML) is one of its most important disciplines. Just as humans can make accurate judgments in many situations by utilizing accumulated experience, ML improves the performance of systems through computation, using “experience” or data. A concise definition of ML is “A computer program is said to learn from experience E with respect to some class of tasks T and performance measure P if its performance at tasks T, as measured by P, improves with experience E” [127]. Task T is how to deal with samples, which refers to the collection of quantized features collected from some objects or events that are expected to be processed by the ML system. The set of samples used to train the ML system is called the training set, and the set used to evaluate the performance of the system is called the test set. Based on the different types of experience E, ML can generally be divided into two categories: supervised learning, where the training data have manually labeled targets, further divided into classification and regression depending on whether the predicted values are discrete or continuous, respectively; unsupervised learning, where the training data do not have labels. Additionally, common ML tasks include transcription [128], [129], machine translation [130], denoising [131], [132], anomaly detection [133], and others.

Machine learning algorithms often require extensive numerical computations, which typically involve algorithms that iteratively update estimates of solutions to mathematical problems. The majority of learning algorithms involve some form of “optimization,” which involves changing the input *x* under certain conditions to achieve the maximum or minimum value of a function *L*(*x*). This function is referred to as the objective function. Maximizing *L*(*x*) can be seen as minimizing −*L*(*x*), so the term “optimization” is often used to refer to the problem of minimizing *L*(*x*), which is also known as the cost function, loss function, or error function in different works. In this context, we provide a brief introduction to some mathematical concepts to enhance the reader’s understanding of the essence of learning algorithms.

**Figure 3:**
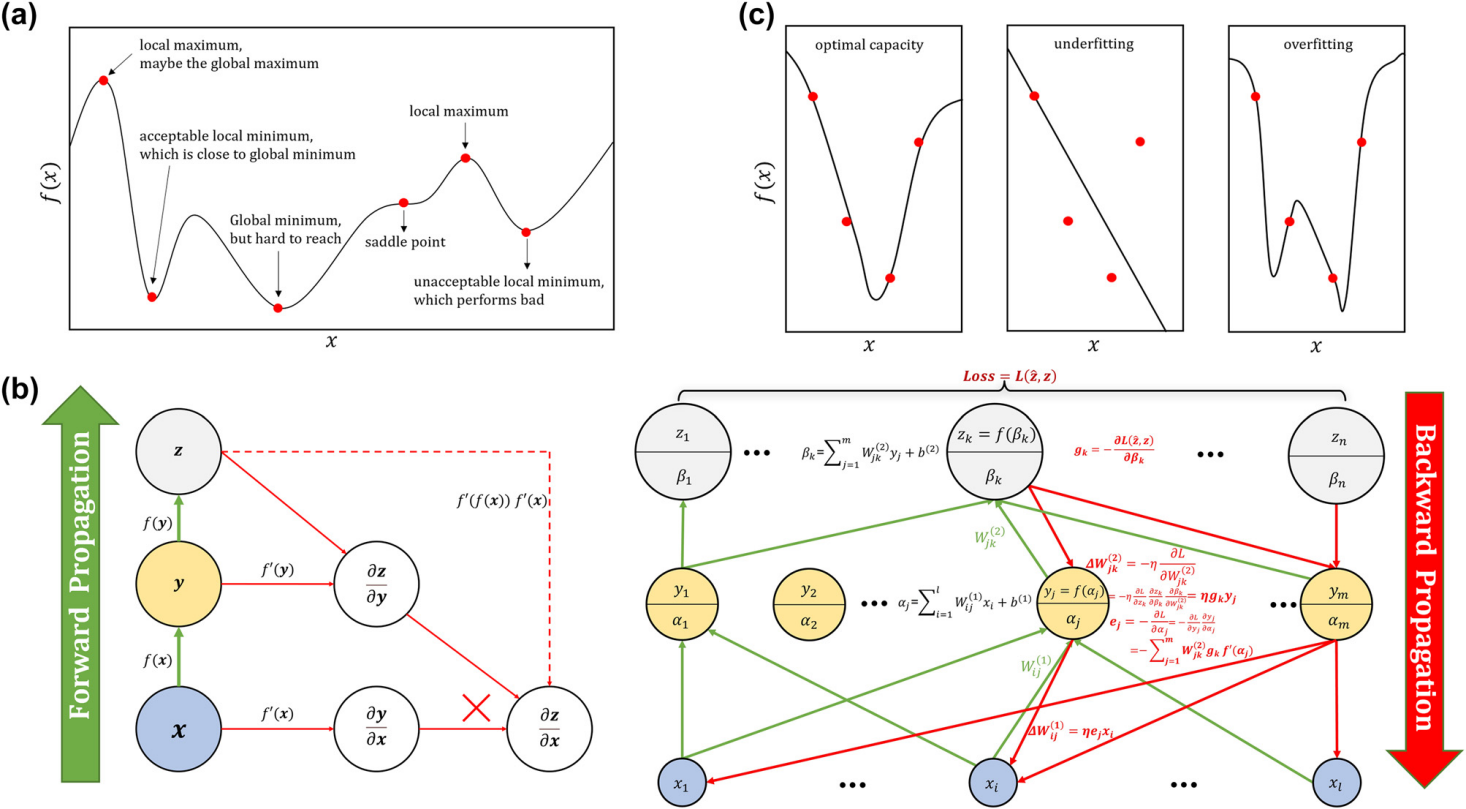
Important mathematical foundation of ML. (a) Different types of critical points. A local minimum means the value of *f*(*x*) is less than all adjacent points, while a local maximum is just the opposite. And there are both larger and smaller neighboring points around saddle point. (b) Computational graph of back propagation algorithm. The left one is a symbolic representation, which just show how to describe the calculation of derivative by symbols. The right one is a more general algorithm flow chart. In order to ensure the clarity of the image, we omitted some of the connected arrows. The green arrows are forward propagation to calculate output *z* through input *x*, and the red ones are back propagation, which calculate the gradient and flow them forward and update parameters. (c) Different models to fitting samples. Underfitting model can’t capture the correct information, while overfitting model provides more false information.

The derivative d*y*/d*x* of a function *y* = *f*(*x*) represents the slope of *f*(*x*) at point *x*. It can be interpreted as the change in the function value when *x* undergoes a small perturbation *δ*: *f*(*x* + *δ*) = *f*(*x*) + *δf*′(*x*). Moving *x* in the opposite direction of the derivative by a small step can decrease *f*(*x*). This technique is known as gradient descent. When the derivative is 0, we cannot determine the direction in which *x* should move. As shown in Figure 3(a), different types of critical points exist in such cases. The ideal solution to the minimization problem is to find the global minimum point. Convex function optimization [134] may be the most successful specific optimization field because convex functions do not have saddle points, and the local minimum point is necessarily the global minimum. However, in general AI learning algorithms, the function to be optimized may have local minima that are not optimal, or there may be many saddle points, especially in the case of multidimensional inputs. For multidimensional functions, the gradient is the derivative of the relative vector, the gradient of a function *f*(*x*) is a vector that contains all the partial derivatives, denoted as ∇_*x*_ *f*(*x*). Moving in the negative direction of the gradient can decrease *f*(*x*). There are many variations of the gradient descent algorithm, including batch gradient descent [135], stochastic gradient descent [136], [137], and mini-batch gradient descent [138], [139].

There are many ways to compute the gradient, among which the most fundamental one in the fields of ML and even deep learning (DL) is the backpropagation algorithm. In learning algorithms, the gradient we most need is the gradient of the loss function with respect to the parameters. Backpropagation is often misunderstood as only being used for multilayer neural networks, but it can actually compute the derivative of any function. We can use a computational graph to represent the algorithm logic simply and describe derivatives with symbols. As shown in Figure 3(b), each node represents a variable, which can be a vector, matrix, tensor, or any other type. At the same time, we need to introduce the concept of “operation,” which refers to a simple function of one or more variables. When multiple operations and variables are combined, more complex functions can be described, and forward propagation calculates the output *z* produced by the input *x*. The difference between the target value $\hat{z}$ and *z* is represented by the loss function $L\left(\hat{z},z\right)$ . To produce the optimal solution, the backpropagation algorithm is executed to update the parameters. By recursively using the chain rule, the gradient can be calculated directly. The three-layer structure shown in the figure only requires two gradient calculations, but as the number of layers increases, the number of repetitions of the required subexpressions will exponentially explode. Compared to calculating the gradient directly, backpropagation reduces the storage requirement. A more general form of backpropagation is shown in the right Figure 3(b), where we introduce activation functions within each layer, allowing it to be extended to nonlinear conditions.

A good ML algorithm has two characteristics: firstly, it performs well on the training set, meaning the training error is small enough; secondly, it performs well when facing unseen new data, meaning the difference between the test error and the training error is small. This feature is also known as generalization performance. These two characteristics correspond to the two main challenges of ML: underfitting when the model cannot achieve a low enough training error on the training set, and overfitting when the model has a large gap between the training error and the generalization error [140]. The principle of Occam’s razor is an ancient philosophical idea that suggests that if there are multiple hypotheses that can explain known phenomena, we should choose the simplest one. This idea guides physicists to continuously propose simple and elegant theoretical formulas and tells us how to improve the generalization performance of ML. Model capacity refers to the ability of a model to fit various functions and can be simply understood as the number of function sets in the hypothesis space. The size of capacity can be changed by altering the number of input features and introducing the corresponding parameters. Obviously, as shown in Figure 3(c) models with low capacity cannot solve complex tasks, while models with high capacity may fall into the trap of overfitting. A simple algorithm is more likely to achieve generalization, but we should also set up sufficiently complex hypotheses to reduce training error. The balance point between the two is difficult to quantify through theoretical analysis and requires continuous exploration and improvement. Regularization is another way to control model capacity. Compared to controlling the number of functions, when facing specific ML tasks, we should control the types of functions allowed to be used. Within the range of samples we care about, there are “naturally” better algorithms than others, which are determined by sample types and data distributions. Therefore, we have preferences for these algorithms, which artificially reduce the generalization error. Optimization and regularization are the most important and central issues in the field of ML [141].

This section starts from the most fundamental mathematical ideas and characterizes and interprets some important concepts in AI and ML. Through the discussions above, we can now have an understanding of how AI can contribute to the research on metasurface. For the various challenges encountered in metasurface research, we can mostly find methods to improve them using AI through corresponding data processing techniques. The following sections will showcase specific AI algorithms and present typical cases of how AI facilitates advancements in metasurface.

## 3 AI for metasurface design

ML provides a data-driven approach that establishes a connection between physical responses and collected data based on manually acquired data [142]. As mentioned before, solving Maxwell’s equations using methods like FDTD and RCWA can be computationally expensive, especially as the number of parameters increases. However, ML can rapidly learn the correspondence between inputs and outputs without being constrained by physical formulas. Generally, with a sufficient amount of data (which often needs to be proportional to the number of parameters), the models generated by ML can achieve accuracy comparable to finite element solvers and can easily be extended to more scenarios. Dramatic advances in machine learning have provided new design methods, metrology, and functionality for nanophotonic devices and systems [143].

This section presents a series of case studies on AI-assisted metasurface design, covering a wide range of ML algorithms and methods, including traditional supervised and unsupervised learning approaches, as well as the increasingly popular deep learning techniques. In the traditional metasurface design process, full-wave simulation is mostly used to calculate parameters such as amplitude and phase of meta-atoms. This is based on a simple assumption: each meta-atom is part of an infinite array of the same structure, and its response originates from near-field coupling from the same neighbors [144]. However, in real situations, since each meta-atom is surrounded by different meta-atoms, the near-field coupling effect will produce deviations, and these radiative coupling [145] or dipole coupling effects will have a huge impact on the overall performance of the metasurface. AI starts from the data and directly considers the meta-atom response after the coupling effect is generated. Not only does it improve the accuracy of calculations in various situations, it can also be compared with assumptions and in turn calculate coupling effects, so that researchers can better understand the physical mechanism of metasurfaces [100]. These cases demonstrate the potential application of ML in metasurface design, providing researchers and engineers with powerful tools and methods to optimize and improve the performance and design process of metasurfaces. By exploring the application of different algorithms, we can gain deeper insights into the development trends of ML in the field of metasurfaces, offering guidance and inspiration for future research and applications.

### 3.1 Classical machine learning

#### 3.1.1 Supervised learning

Among all machine learning algorithms, one of the most well-known is Support Vector Machine (SVM) [146]. It is designed for binary classification tasks. Given a sample training set *D* = {(*x*, *y*)}, the classification problem is to find a hyperplane that separates samples of different classes. This separating hyperplane can be described by a linear equation ***w*** ^***T***^ ***x*** + *b* = 0, where ***w*** is the normal vector and *b* is the distance term. Assuming it can correctly classify the training samples, the feature vectors corresponding to the samples closest to the hyperplane are called support vectors, and the sum of the distances from two support vectors of different classes to the hyperplane $\gamma =\frac{2}{\left\|w\right\|}$ is called the margin. The basic idea of SVM is to maximize the margin, which means finding the classification hyperplane with the best robustness and generalization performance. Therefore, its basic mathematical form can be represented as

$$\underset{w,b}{\max}\frac{2}{\left\|w\right\|}$$

$$\text{s}\cdot \text{t}\cdot \;y_{i}\left(w^{T}x_{i}+b\right)\geq 1,i=1,2,\ldots .,m$$

This training process is a convex optimization problem.

**Figure 4:**
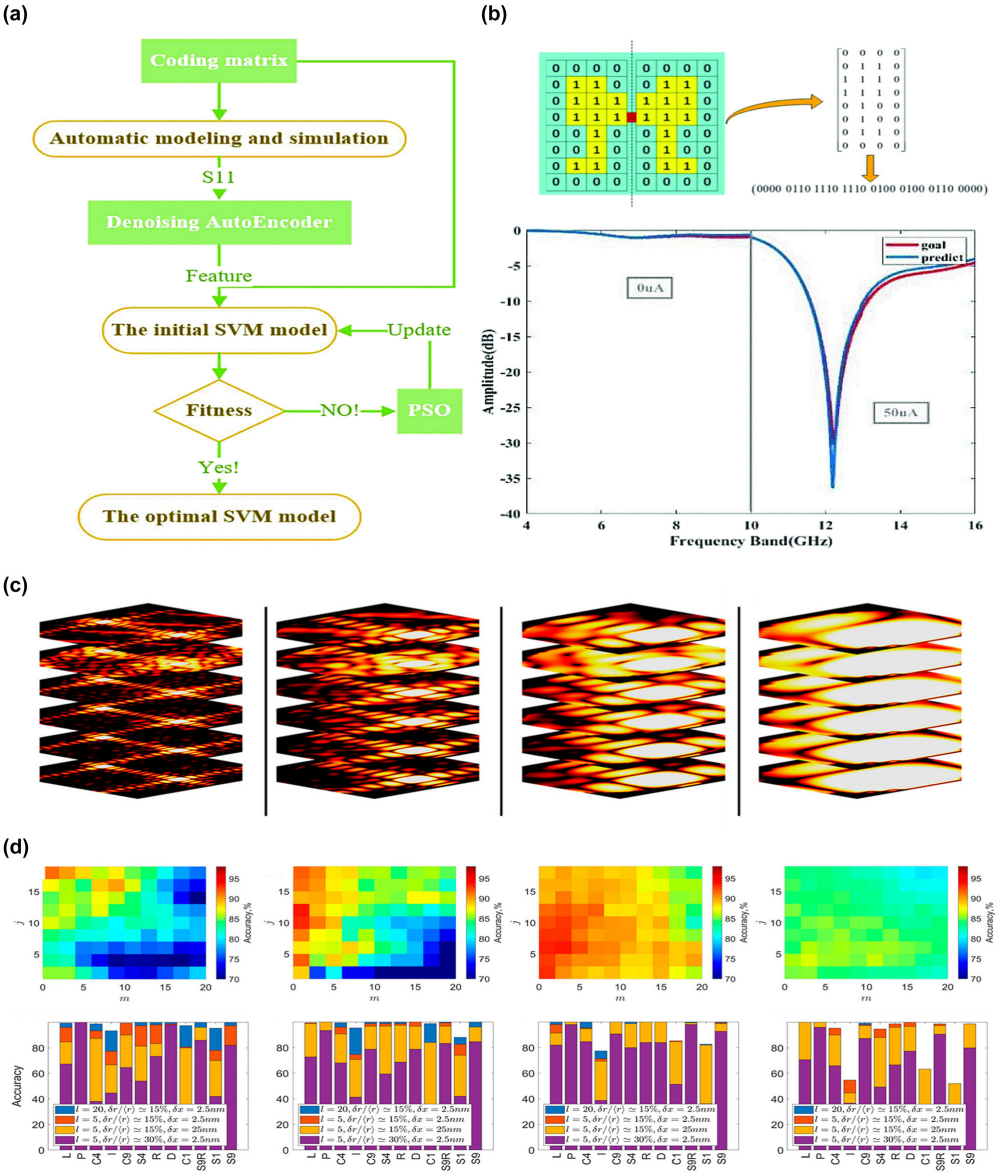
SVM for metasurface design. (a) Flowchart of reconfigurable metasurface design based on SVM model. Reproduced from [147]. Copyright 2022, IEEE. (b) Above is coded reconfigurable metasurface. Below are target amplitude curve (red) and predicted amplitude curve (blue) of 1 bit amplitude modulation metasurface. Reproduced from [147]. Copyright 2022, IEEE. (c) Diffractive signatures generated theoretically for free-space wavelength of 532 nm, 1 μm, 2 μm, and 4 μm. Reproduced from [148]. Copyright 2021, American Chemical Society. (d) Accuracy of the SVM classifiers, and classification accuracy of the particular object for different wavelength in (c). Reproduced from [148]. Copyright 2021, American Chemical Society.

Due to its good generalization ability and prediction accuracy, SVM has been widely used in metasurface design problems [147], [148]. In practical cases, since the training samples are not necessarily linearly separable, which means there may not exist a hyperplane in the original sample space that can correctly classify them, the kernel function method is used to map the original sample space to a high-dimensional feature space, making the training samples linearly separable in the new space. Lu et al. [147] proposed a fast reconfigurable metasurface design method based on SVM, which was used to predict the amplitude curves under different structures. The algorithm framework is shown in Figure 4(a). They designed a two-dimensional QR code-like metasurface structure, which is symmetric about the *Y*-axis and each region has 8*4 pixels. The pixels are encoded with “1” and “0” to indicate whether they are filled with metal. To train the SVM model, they generated 10,000 structures and calculated the corresponding electromagnetic characteristics using CST. For each of the 32 pixel regions, they built a separate SVM model, mapped the electromagnetic response to a high-dimensional space using the Gaussian kernel function [149], [150], and introduced PSO to optimize the penalty coefficient *C*, which denotes the tolerance of error, and the kernel function parameter *γ*, which affects the speed of training and prediction [151], [152].

To verify the capability of the SVM model, a metasurface resonating at 6.17 GHz was designed, demonstrating good predictive performance, showed in Figure 4(b). In general, reverse identification of metasurface structures based on diffraction patterns is quite challenging, especially when the feature differences between most samples are small. In such cases, SVM can assist in the classification and identification tasks of complex images. Ghosh et al. [148] introduced a SVM-based classifier to analyze a diffraction image library parameterized by specific combinations, thereby identifying the subwavelength-level unit features carried by the metasurface. Figure 4(c) shows the diffraction images in free space with incident wavelengths of 532 nm, 1 μm, 2 μm, and 4 μm, respectively. Once the classifier is trained, the identification process can be performed based on the diffraction pattern measured in a single experiment. The researchers represented the intensity of diffraction images in the form of Bessel transforms:

$$I\left(k_{r},\phi \right)\approx \underset{m,j}{\sum }C_{m_{j}}J_{m}\left(\alpha_{m_{j}}\frac{k_{r}}{k_{0}}\right)\cos\left(m\phi \right)$$

where the indices *m* and *j* describe the distribution of intensity radially on the angular angle. The SVM classifier is used to pair the combinations of parameters (*m*, *j*) and analyze the accuracy of the generator. The final results are shown in Figure 4(d), indicating that the proposed ML-based detection technique can identify nanoscale units at the 1/5 wavelength level. This approach can be used not only for characterizing metasurface with resonant elements in reflection or transmission modes but also for reverse designing metasurface based on desired diffraction images. Based on the classification problem, the Support Vector Regression (SVR) algorithm has been developed for regression problems. It applies kernel functions to select hyperplanes that include the maximum number of data points, thereby predicting numerical outputs in regression problems [153], [154].

k-Nearest Neighbor (kNN) algorithm is also one of the simplest ML algorithms that is applicable to both classification and regression problems [155]. It has been successfully utilized in antenna design and electromagnetic field optimization. The principle behind kNN is to find the *k* nearest training samples in the training set to a given test sample based on a certain distance measure and use them to predict new data. In regression problems, kNN calculates the average or weighted average of the outputs of the nearest points, which can be represented as $\hat{y}=\frac{1}{k}\overset{k}{\underset{i=1}{\sum }}y_{i}$ .

**Figure 5:**
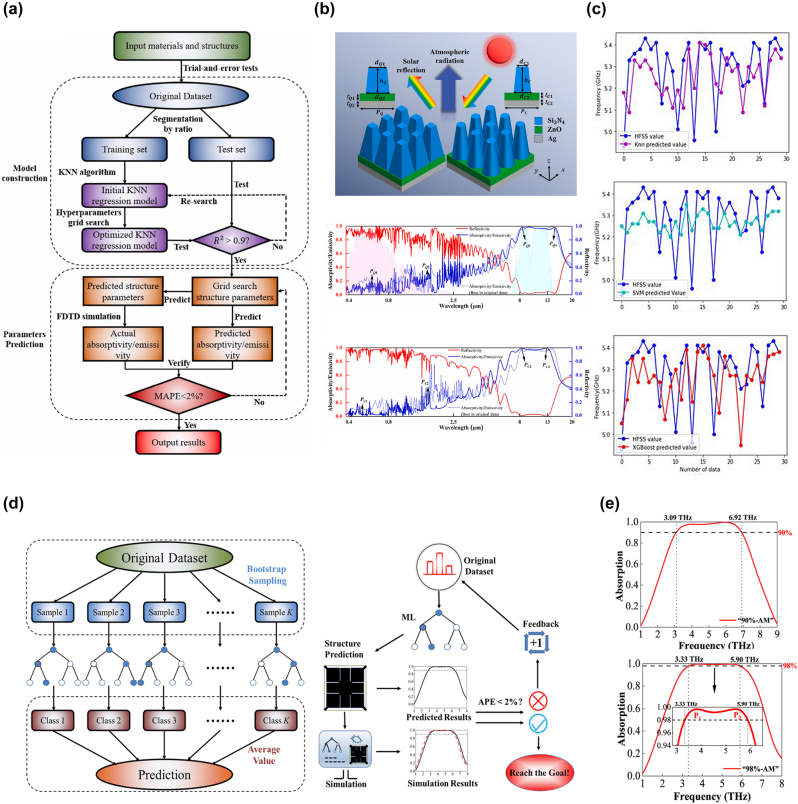
kNN and RF for metasurface design. (a) Flowchart of the radiative cooler structural parameters prediction combined with kNN regression model. Reproduced from [156]. Copyright 2023, Elsevier. (b) The proposed QPM and CTCM units and the absorptivity/emissivity spectrum (including the best in original data) and reflectivity spectrum. Reproduced from [156]. Copyright 2023, Elsevier. (c) Simulated and predicted resonance frequency of kNN, SVM, and XGBoost versus HFSS. Reproduced from [157]. Copyright 2022, IEEE. (d) Flowchart of the process to predict metasurface structural parameters combined with RF generation process [160]. (e) Absorption spectra obtained from FDTD simulation of the predicted parameters for “90%-AM” and “95%-AM” [160].

Unlike SVM, kNN follows a lazy learning approach where it only stores the samples during the training process and performs computations when a test sample is given. Su et al. [156] proposed an optimization method for daytime radiative cooling using metasurface based on kNN. They employed kNN to construct a regression model for optimizing the structures of quadrangular prismatic metasurface (QPM) and circular truncated cone metasurface (CTCM), as shown in Figure 5(a) and (b). The metasurface consists of two layers: Ag and ZnO as the substrates and Si_3_N_4_ structures. Four variables, including the upper and lower diameters of Si_3_N_4_ pillars, the height of the pillars, and the thickness of the ZnO layer, were selected as the feature variables for predicting the absorption/emission rates. They calculated 500 different structures, with 90 % of them used as the training set for the kNN regression model. After training, the R-squared values for all predicted values in the regression model were above 0.9, and the mean absolute percentage errors (MAPE) for the two structures were only 0.74 % and 0.29 %, respectively. Based on this, optimization design was conducted for the two structures, resulting in absorption/emission spectra that reduce in the solar spectrum and increase in the atmospheric window. Additionally, the optimized structures exhibited polarization insensitivity and angle independence, demonstrating ideal characteristics. Similarly, Nuzhat et al. [157] utilized a kNN-based machine learning model to predict the resonant frequencies of Artificial Magnetic Conductor (AMC) cells and compared it with other ML algorithms. They inputted the structural parameters L and G and simulated 129 sets of resonance curves using ANSYS HFSS as the dataset. During kNN training, they selected *k* = 3 as the optimal value. The results are shown in Figure 5(c), the trained kNN model exhibited good consistency with HFSS and required much less time and computational resources than traditional electromagnetic simulation methods. Despite its simplicity, kNN demonstrated excellent performance in predicting AMC resonance frequencies due to its ability to handle small-dimensional datasets with a large number of measurement values. In contrast, SVM showed larger prediction biases as it is not suitable for datasets with small dimensions and a large number of measured values. On the other hand, XGBoost [158], known for its speed and based on decision tree boosting, showed comparable or even superior capabilities to kNN.

In addition to SVM and kNN, another widely used ML algorithm for classification and regression is Random Forest (RF). RF involves processes such as random bootstrapping, decision tree construction, feature variable selection, node splitting, and ensemble prediction. It constructs multiple decision trees and integrates their predictions through voting or averaging to achieve high-performance classification and regression tasks [159]. Ding et al. [160] proposed a method using the RF algorithm to design patterned graphene metasurface absorbers. The design process, as shown in Figure 5(d), addresses the complex impedance matching and field excitation involved in the electromagnetic absorption process of patterned graphene metasurfaces. It is difficult to determine the effects of various parameters. By treating the structural parameters of patterned graphene as feature variables, RF effectively minimizes the correlations between decision trees through randomness, thereby improving the accuracy of predicting absorption parameters. Based on the trained RF model, the predicted maximum effective absorption bandwidth and maximum perfect absorption bandwidth were 3.77 THz and 2.54 THz, respectively, along with the corresponding structural parameters. Figure 5(e) shows the FDTD simulation verification based on the predicted parameters, resulting in bandwidths of 3.83 THz and 2.57 THz, with a MAPE of only 1.36 %. This demonstrates the sufficient predictive accuracy of the RF regression model in the design of patterned graphene metasurfaces. Additionally, to showcase the superiority of RF, they also built other classical ML regression models, including linear regression (LR), SVM, least squares (LS), and kNN. Only RF achieved an R-squared error of above 0.9, while the remaining models had an average value of only 0.78. Compared to the previous two algorithms, RF is more suitable for handling high-dimensional and large-scale datasets, and it possesses good generalization ability and robustness. This is due to the ensemble learning framework on which RF is based, where individual decision tree predictions can often be corrected by other decision trees, and it is not sensitive to parameter selection and noise influences.

#### 3.1.2 Unsupervised learning

The mentioned algorithms are all supervised learning algorithms, where the training dataset consists of input samples along with their corresponding labels or outputs. The goal of these algorithms is to establish a relationship between the inputs and outputs to make predictions for unseen inputs. On the other hand, unsupervised learning involves only input samples without corresponding labels, and the objective is to discover inherent structures, patterns, or features within the data. Common unsupervised learning tasks include clustering [161], [162], density estimation [163], [164], anomaly detection [133], [165], and more.

**Figure 6:**
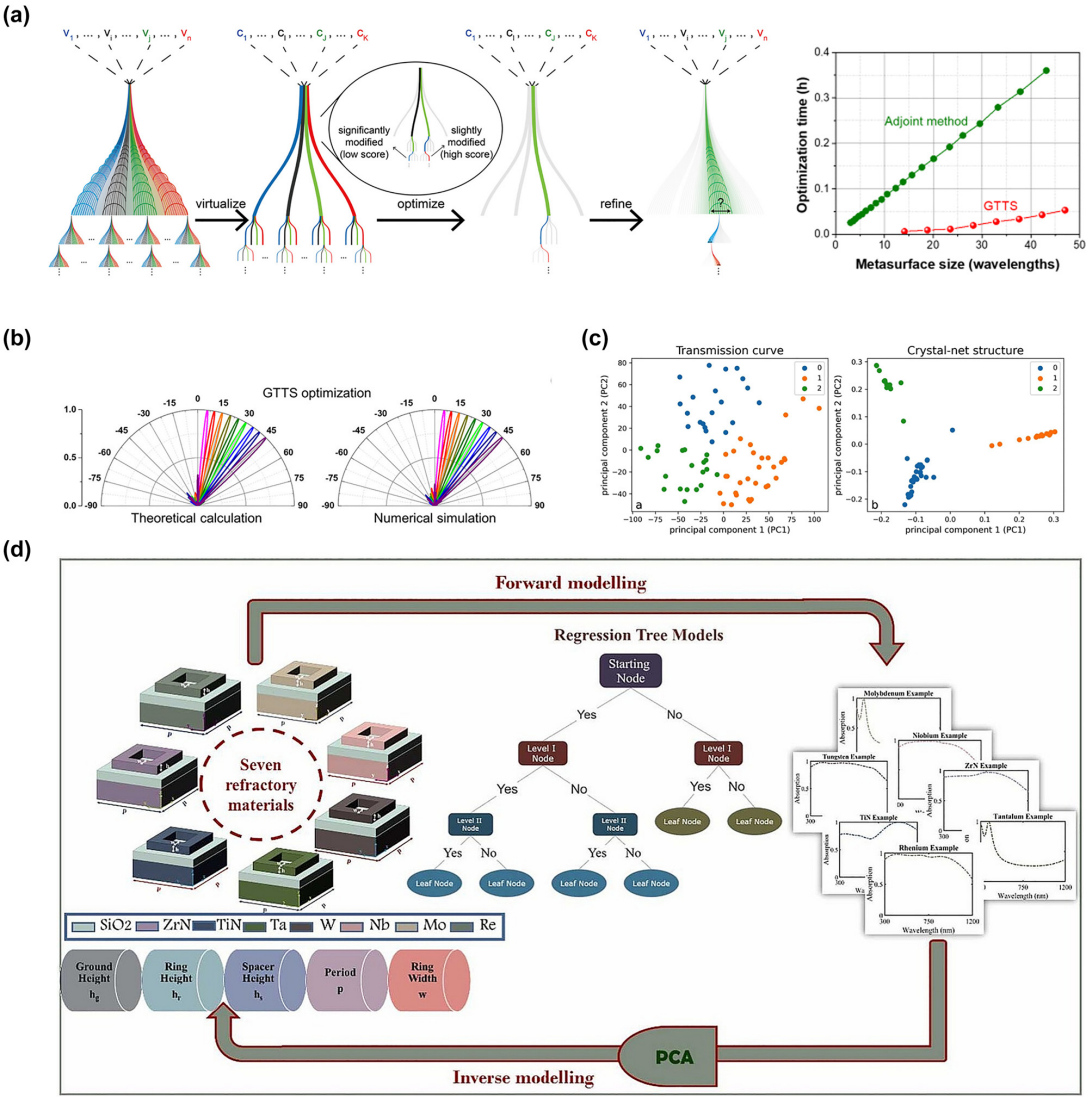
Classical unsupervised machine learning for metasurface design. (a) Flowchart of GTTS combined with unsupervised clustering, and comparison of time cost of adjoint method and GTTS. Reproduced from [166]. Copyright 2021, American Chemical Society. (b) Comparison of normalized far-field radiation intensity versus steering angle obtained from theoretical calculation and numerical simulation. Reproduced from [166]. Copyright 2021, American Chemical Society. (c) Results of K-means clustering using transmission curve and radial distribution function. The coordinates are calculated by PCA [167]. (d) Flowchart of seven refractory material meta-absorber design. Forward model shows the absorption prediction using regression tree algorithm while inverse model utilizes PCA before determining the design parameters. Reproduced from [168]. Copyright 2023, Elsevier.

Lin et al. [166] proposed a genetic-type tree search algorithm (GTTS) combined with unsupervised clustering for the automatic inverse design of metasurfaces that enable high-directional beam steering. The problem of specifying the number of nanoscale antennas and optimizing their arrangement to achieve the desired far-field radiation pattern is a nondeterministic polynomial hard (NP-hard) combinatorial problem. It is a nonconvex optimization problem. To address this, they applied the GTTS algorithm and introduced the concept of virtual space for efficient optimization, as shown in Figure 6(a). First, they constructed a database *V* containing n nanoscale antennas. By using existing k-means solvers and unsupervised SVM, the antennas were clustered into *K* groups. Nanoscale antennas within the same group exhibited different but similar physical characteristics. The k-means solver also outputted a center-average structure *C*, which represents the average of all similar nanoscale antennas and can represent the entire group. Therefore, the search was performed only on the library with *K* virtual nanoscale antennas, and the resulting metasurface was similar to searching the entire library. Then, refinement was applied to each unit to transform the optimal virtual design back to the original space. Compared to commonly used adjoint optimization, the GTTS method significantly reduced the computational time. The original structure yielded nonintuitive amplitude and phase distributions. By utilizing GTTS, both parameters could be simultaneously optimized to deflect the incident light to the target angle. To further validate the optimization results, numerical simulations were conducted, which showed the far-field radiation intensity of nanoscale antennas with the same arrangement. The simulations considered near-field coupling, while the theoretical calculations treated the nanoscale antennas as independent dipole sources. The agreement between the two approaches confirmed the assumption of neglecting internal coupling during the GTTS optimization process. The researchers successfully designed a metasurface with high-performance active beam steering capability. Figure 6(b) illustrates the relationship between the normalized far-field radiation angle and the steering angle for the designed electrically tunable metasurface in the mid-infrared range.

Many ML training processes are based on an important assumption: the sampling density of the training samples is sufficiently high, known as the Nyquist sampling condition. However, as the dimensionality of the attributes increases, the number of samples required to satisfy the Nyquist sampling condition grows exponentially. Distance-based methods, such as SVM, encounter significant challenges in high-dimensional situations. The problems of sparse sample data and difficult distance calculations in high-dimensional spaces are common obstacles faced by all ML methods and are referred to as the “curse of dimensionality.” Although the curse of dimensionality is widespread, it is not unsolvable. In the field of design optimization for nanophotonic devices, although the collected data are high-dimensional, only a low-dimensional distribution relevant to the learning task may exist, known as a low-dimensional embedding in the high-dimensional space. Therefore, data can be processed through dimensionality reduction techniques.

Principal Component Analysis (PCA) is a classical dimensionality reduction method that has been widely used even before the advent of computers. Based on maximum separability, if the projections of all sample points in the high-dimensional orthogonal space onto a low-dimensional hyperplane can be maximally separated, the variance of the projected sample points should be maximized. Given the dimensionality d of the low-dimensional space and the sample set, the main idea of PCA is to decompose the covariance matrix of normalized samples into eigenvalues and select the eigenvectors corresponding to the largest d eigenvalues as the solution. Obviously, the low-dimensional space differs from the original high-dimensional space because some eigenvectors are discarded, but discarding this part of information increases the sampling density of the samples and also acts as a denoising effect. Hou et al. [167] introduced the reticular chemistry structure resource (RCSR) to design meta. They used PCA and the k-means algorithm to visualize and analyze the transmission curves of 72 metasurfaces. The results showed that the transmission curves could be classified into three main types, but a simple relationship between structure and transmission rate had not been found, as shown in Figure 6(c). This design approach provides a novel “alternative” method to modify the transmission curves of metasurfaces without focusing on adjusting the structural parameters of individual atoms. Ijaz et al. [168] developed multiple forward regression models in Figure 6(d), including Decision Tree Regressor (DTR), kNN, LR, and Polynomial Linear Regressor (PLR), to predict the absorption values of seven different materials. The dataset consisted of 16,807*7 responses, and to compensate for the data processing load, PCA was used to preserve the integrity of the data structure and eliminate redundancy by projecting the high-dimensional information onto a lower-dimensional subspace.

There are many unsupervised learning algorithms that can be applied to metasurface design, such as autoencoders based on neural networks, which will be discussed in the next section. Various algorithms, their variations, and combinations are beyond the scope of this discussion, but interested readers can refer to machine learning–related literature for more information [169], [170], [171].

### 3.2 Deep learning

#### 3.2.1 Supervised learning

The performance of classical ML algorithms largely depends on the representation, also known as features, of the given data. The core of many AI tasks is to extract a suitable set of features and provide them to simple ML algorithms. However, when facing complex real-world tasks, it is difficult to determine which features should be extracted due to the different perceptions of humans and computers. Representation learning is a way to address this issue by using machine learning to discover representations themselves, rather than just mapping representations to outputs. The learned representations are often better than manually designed ones and require minimal human intervention to adapt quickly to new AI tasks. Deep learning (DL) is a prominent approach in representation learning, which expresses complex representations through simpler ones, allowing computers to represent complex concepts using simpler concepts [172]. DL is essentially a branch of ML but is well-known for its use of Artificial Neural Networks (ANNs). DL utilizes hierarchical structures to learn multiple levels of data abstraction, providing an efficient method for designing photonic structures [173]. The following paragraphs will introduce the applications of several commonly used DL models in metasurface design.

**Figure 7:**
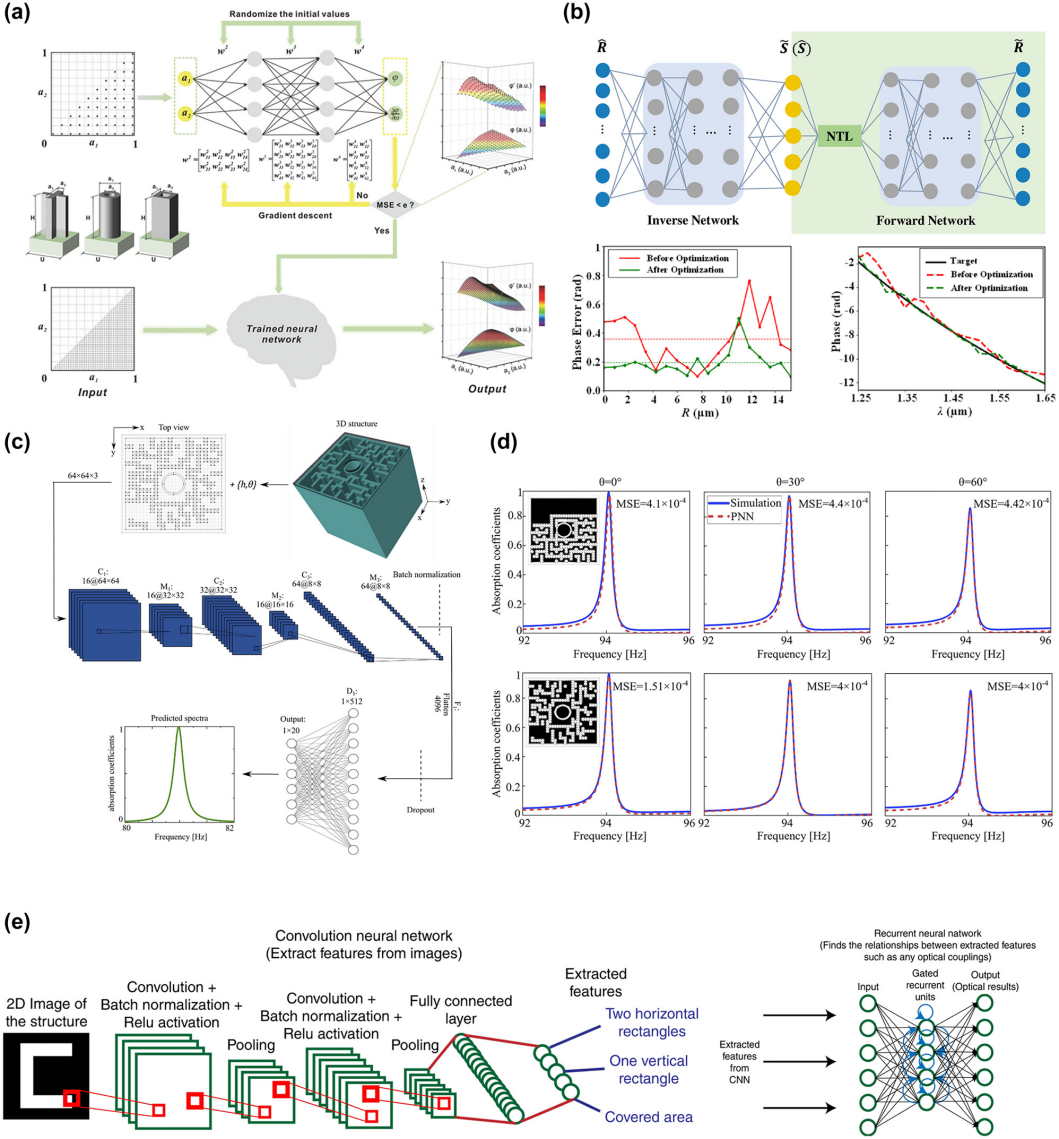
Supervised ANN for metasurface design. (a) Flowchart of the backpropagation neural network for predicting phase response and phase gradient. Reproduced from [176]. Copyright 2022, Wiley Materials. (b) Schematic of tandem neural network. The lower left shows the phase errors before (red) and after (green) DNN optimization. The lower right is an example of the target fitting method. Reproduced from [177]. Copyright 2021, Optica Publishing Group. (c) Flowchart of the CNN-based predicting neural network for metasurface absorber design. Reproduced from [180]. Copyright 2022, Elsevier. (d) Comparison of the predicted absorption spectra (red) and simulated absorption spectra (blue). Reproduced from [180]. Copyright 2022, Elsevier. (e) Flowchart of the combined deep network. CNNs are used to extract spatial data from smaller parts of the image while RNNs are used to find the required optical response based on the features extracted from CNNs [184].

Supervised deep learning models, often referred to as discriminative models in the context of metasurface design, include the basic Artificial Neural Network (ANN) called the Multiple Layers Perceptron (MLP) [174]. MLP is composed of multiple layers of nested nonlinear functions, where each layer is a nonlinear function of the form *h* = *g*(*W* ^*T*^ *x* + *b*). Here, *x* is the input vector, *W* and *b* are the weight and bias parameters, and *g* is the activation function. In theory, an MLP with a sufficient number of layers can approximate any nonlinear function [175]. Figure 7(a) demonstrates a backpropagation neural network proposed by Wang et al. [176] for predicting the phase and phase gradient of meta-units with different structural parameters. An ideal achromatic lens should possess a dispersed phase distribution $\varphi \left(r,\omega \right)$ . By considering the focal shift effect, the phase can be expressed as $\varphi \left(r,\omega \right)=-\frac{\omega_{0}}{c}\sqrt{r^{2}+k^{2}\times {\omega_{0}}^{-2}}+\alpha {\omega_{0}}^{2}+\beta \frac{\omega_{0}}{c}+\gamma$ and the phase gradient is defined as $\varphi^{\prime }={\left.\frac{\partial \varphi }{\partial \omega }\right|}_{\omega =\omega_{0}}=\frac{-r^{2}}{c\sqrt{r^{2}+k^{2}\times {\omega_{0}}^{-2}}}+2\alpha \omega_{0}+\frac{\beta }{c}$ , which is based on the radius (*r*). They designed three styles of meta-atoms: a cross, a hollow circle, and a hollow square. The height and period values were fixed, and the overall structure was described by only two parameters. Therefore, the input and output dimensions of the network were both 2. They trained the network using only 695 data samples, and the network generated over 15,000 sets of structure-phase data within 0.67s. Based on the predicted data, they designed a chromatic aberration-free metalens for the 420–640 nm bandwidth. Although the phase response and meta-atom size have a highly nonlinear relationship, a neural network with a small input and output dimension, such as an MLP with two hidden layers, can accurately describe their relationship. This approach alleviates the computational burden. An et al. [177] designed a tandem network model, as shown in Figure 7(b), which consists of two MLPs connected together. The forward network is used to fit the input meta-unit parameters $\tilde{S}$ and the output EM response $\tilde{R}$ , while the inverse network predicts the parameters $\hat{S}$ from the desired EM response $\hat{R}$ . They also introduced a Neural Tensor Layer (NTL) to match the dimensions between the inputs and outputs. Two datasets were collected for two different structures, and the networks were trained using the Mean Squared Error (MSE) loss function. After 500 epochs, the MSE values were 0.098 and 0.147, respectively, which closely matched the responses obtained through FDTD calculations, demonstrating the network’s ability to predict electromagnetic responses. Although MLPs have demonstrated strong fitting capabilities, there are still limitations. For instance, MLPs are not suitable for models with excessively large or extremely mismatched input and output dimensions. Additionally, MLPs do not provide a fully automated optimization process and require adjusting the network structure and hyperparameters based on experience [178].

Most metasurfaces are two-dimensional structures, which mean that the structure of a meta can be viewed as image data in a 2D pixel grid. Convolutional Neural Network (CNN) is a type of neural network specifically designed to handle data with grid-like structures [179]. The basic structure consists of three layers: the convolutional layer performs convolutional operations on the input 2D image using kernel functions, generating a set of linear activation responses to extract image features. The second layer is the detector layer, where each linear activation response passes through a nonlinear activation layer. Finally, the pooling layer replaces the network’s output at a particular position with aggregated statistical features of neighboring outputs, adjusting the output of the detection layer. CNN can be seen as an MLP with infinitely strong priors, indicating that the learned functions at each layer only contain local connectivity relationships. Additionally, the pooling layer serves as a prior that introduces translational invariance to the input. Compared to MLP, CNN has fewer trainable parameters. Donda et al. [180] constructed a CNN-based Prediction Neural Network for the forward design of metasurface absorbers, predicting the absorption spectrum based on the structure of the metasurface. To facilitate the learning process of the neural network, they initially processed the absorption spectrum data by using PCA to reduce the dimensionality from 201 to 20. As shown in Figure 7(c) and (d), the surface of the metasurface was transformed into a binary image of 64 × 64 pixels, and additional channels for thickness and incident angle were included as inputs. After passing through three convolutional-pooling layers, batch normalization, and dropout layers, the network outputted a spectral curve with 20 points, achieving an MSE of only 0.00904. Subsequently, an ablation analysis was conducted to demonstrate the rationality of the network parameters. Additionally, they constructed a CGAN-based network architecture for inverse design, which could predict the metasurface structure with specified absorption spectrum and thickness given the incident angle. The final designed metasurface absorber had a thickness of only *λ*/64 and exhibited nearly omnidirectional absorption performance at 82 Hz. CNN is often used in combination with other networks such as Recurrent Neural Networks (RNNs) [181], Residual Networks (ResNets) [182], and U-net [183]. Figure 7(e) illustrates the combined neural network model proposed by Sajedian et al. [184]. The model consists of a CNN with residual connections and a small-scale RNN. ResNet incorporates multiple layers of CNN, where the output of a particular layer is added to the output of the next layer. This enables gradient backflow to earlier layers, allowing for the design of deeper networks without encountering vanishing gradients. As the model deepens, it extracts higher-level features. They trained the model using 100,000 data pairs and achieved near-perfect predictions for all 10,000 results in the validation set. The final model can predict the optical response of any structure based on an image within 1 s, showcasing the potential of CNN in metasurface inverse design.

#### 3.2.2 Unsupervised learning

**Figure 8:**
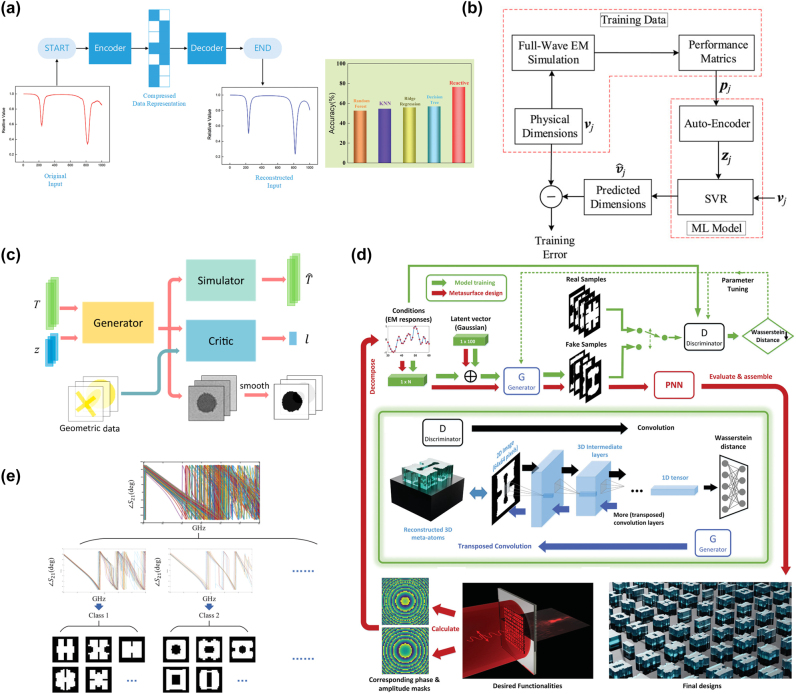
Unsupervised ANN for metasurface design. (a) Under-complete AE in REACTIVE, reducing the number of data dimensions from 1000 to 200. The proposed REACTIVE has higher accuracy compared to other ML models [187]. (b) Flowchart of training process model based on AE and SVR. Reproduced from [188]. Copyright 2023, Wiley Materials. (c) Architecture of the GAN-based optical design. The whole network has three parts: generator, simulator, and critic. Reproduced from [192]. Copyright 2018, American Chemical Society. (d) Flowchart of the generative meta-atom design network based on WGAN. Generator and discriminator approach the ground truth via this adversarial process. After training, translate the desired function to phase and amplitude as the input of generator, then it can generate actual meta-atom arrays. Reproduced from [193]. Copyright 2021, Wiley Materials. (e) kNN-based classification for metasurface cells. The entire sample of metasurface cells is divided into a finite number of classes according to their phase curves. Reproduced from [194]. Copyright 2022, IEEE.

Unsupervised deep learning is contrasted with supervised deep learning, which encompasses generative models [185], [186]. Representative algorithms in this category include Autoencoder (AE) and Generative Adversarial Network (GAN). After training, an AE can copy the input to the output, consisting of an encoder function *h* = *f*(*x*) and a decoder function *y* = *g*(*h*). It is worth noting that AE does not aim to learn the exact solution of *g*(*f*(*x*)) = *x* but rather achieves an approximate replication by imposing constraints. This approach often enables the learning of useful features from the data. These constrained models consider which parts of the data should be prioritized for replication, allowing them to learn useful features of the data. In the metasurface design process, AE is often used for dimensionality reduction, and the learning process can be succinctly described as minimizing *L*(*x*, *g*(*f*(*x*))). When the decoder is linear and *L* represents mean squared error, the AE learns the same generative subspace as PCA. However, if both *f* and *g* are nonlinear, the AE can learn a more powerful nonlinear extension of PCA. Qiu et al. [187] proposed a three-step metasurface design method framework called REACTIVE, where Cepstrum transformation was initially used for feature extraction to reduce the dimensionality of the input metasurface structure from 1000 to 200. Subsequently, an under-complete AE in Figure 8(a) further extracted 64-dimensional features. Compared to other ML algorithms, the REACTIVE method achieved higher accuracy. Ahmed et al. [188] addressed multiple design constraints such as S-parameters, gain, radiation efficiency, and radiation pattern by proposing a maximum likelihood model composed of cascaded AE and SVR. In Figure 8(b), AE was employed for the dimensionality reduction of multiple antenna performance metrics, and the model could predict the design variables of nanoantennas based on the desired performance metrics. AE has a famous variant, which is called variational autoencoder (VAE). This model can realize both the forward prediction of optical responses of a certain structure and the inverse design of structures from preconfigured optical properties [189].

Whether it is forward design or inverse design, human intervention is required for data selection and analysis. However, the existence of GANs breaks through this limitation [190], [191]. Many generative models are based on the idea of differentiable generator networks, which employ a differentiable function *g*, usually a neural network, to transform latent variables *z* into distributions on samples *x*. GANs, proposed based on game theory, consist of two components: the generator, responsible for generating samples $x=g\left(z;\theta^{\left(g\right)}\right)$ , and the discriminator, responsible for judging the authenticity of the samples. The generator aims to deceive the discriminator as much as possible, while the discriminator strives to distinguish samples drawn from the generator from real samples in the training set. They engage in a game to improve performance. Unlike traditional machine learning algorithms, GANs are often difficult to evaluate in terms of loss because the generator and discriminator are fundamentally in an adversarial relationship, and there is no convergence of the loss function. In such cases, alternative methods are required to measure the loss. GANs have been successfully used to construct complete and fully automated optimization design programs, reducing human intervention, and have been proven to be efficient and effective in metasurface design. Liu et al. [192] employed GANs to search for structures that yield the desired input spectra, enabling the network to generate metasurface patterns based on arbitrary input optical responses. The metasurface used for training consist of a single layer of gold, and using finite-element modeling (FEM), the transmission spectra of metasurface with different shapes under x and y polarizations within the visible to near-infrared range were simulated. As shown in Figure 8(c), the structures were simultaneously represented as binary images of 64*64 pixels. The trained generator was able to approximate the transmission rate at each frequency point with an average error of less than 0.01. The model could handle multiple input spectra without sacrificing accuracy and was easily scalable to more complex structures. The work by An et al. [193] utilized a more stable variant of GAN called Wasserstein-GAN (WGAN) and proposed a novel metasurface design approach. By introducing Wasserstein distance as a loss evaluation metric, the training process was stabilized, enabling the network to be widely applicable in metasurface design. In Figure 8(d), both the generator and discriminator are essentially CNNs, as mentioned earlier, capable of capturing the nonlinear relationship between structure and response. A preprocessing layer was added before the first input layer, transforming phase and amplitude responses into complex transmission coefficients. The conditional *x*-target pairs were combined with noise to confuse the discriminator. This network exhibits high stability and ease of convergence. Once the training is completed, for different applications, the desired responses can be input into the generator to design the desired distribution of meta-atoms. They successfully applied this network to design a bifocal lens, a polarization multiplexed deflector, a polarization-multiplexed lens, and a polarization-independent lens. Huang et al. [194] used a combined model of GAN and kNN, where preclassifying the dataset with kNN effectively enhanced the learning capacity of GANs, as shown in Figure 8(e).

In addition, there are emerging ML algorithms such as Reinforcement Learning (RL) [195], Deep Q-Learning (DQN) [196], Transformer [197], and Normalization Flows (NF) [198].

In this section, we have discussed many kinds of AI algorithms. According to the characteristics, advantages, and limitations of each algorithm, we summarize them in Table 1. In practical research, attention needs to be given to the form of the dataset. The input and output dimensions of the dataset vary depending on the structural parameters and target responses of different metasurface. One simple solution is to increase the number of samples in the dataset, as more samples imply more features, which can help improve the training accuracy of ML algorithms. However, in many cases, due to limited sample acquisition or chaotic distribution of data, it is necessary to select suitable ML algorithms for specific problems. Furthermore, relying on experience or other optimization algorithms to adjust the parameters of ML algorithms is often required. This work currently lacks a definitive conclusion because of the “No Free Lunch” theorem, which states that the expected performance of all learning algorithms is the same [199]. Therefore, it is crucial to focus on specific application tasks and the selection of a learning algorithm that matches the problem, as it plays a vital role in research.

**Table 1:** Comparison of different AI algorithms.

Algorithms	Network structure	Input	Output	Dataset size	Runtime	Accuracy	Advantages	Limitations	Ref.
Supervised learning	MLP	GP (2)	Phase/Phase gradient (2)	695	Less than 10s		Simple network structure, small amount of data required, short training time	For simple inputs and outputs only. Not applicable to binarized structures or curve prediction with heavily randomized values	[176]
		GP (5)	Phase curve	24,000		MSE 0.147			[177]
	CNN	2D grid (64*64)	Spectra curve (20)	9000	8 h	MSE 9.04e-3	Most structures can be processed as images, especially for binarized structures.	The network effect is very dependent on the parameter settings. The amount of data required is large, while the training time is long	[180]
		2D grid (100*100)	Absorption curve (1000)	100,000	15d	MSE 4.2591e-5			[184]
	U-net	2D grid (120*120)	Six-channel EM near-field response (120*120)	10,000	6 h	MSE 5.76e-7	Better preserve image information and fuse multiscale information. More lightweight. Data enhancement strategies can improve the generalization ability of the network and mitigate the overfitting problem.	A fixed symmetric network structure is used, which may not perform well for targets with complex or irregular shapes. A large amount of labeled data is usually required to train and optimize the model.	[183]
Unsupervised learning	AE	Extracted feature (200)	Extracted feature (64)	2000	3.5 m	97 % similarity	Useful for tasks such as data visualization, feature extraction, and dimensionality reduction. It can help in tasks such as extending the dataset, generating new samples, and performing data enhancement. The network structure is flexible and the training process can be used as part of pretraining and then the learned features can be migrated to other related tasks.	There are no labels or supervisory signals to assess model performance. Rely on other metrics or by comparing performance with other tasks. Often makes certain assumptions about data distribution, which may limit performance when dealing with complex data distributions and outliers. May have degraded performance when dealing with nonsmoothed or discontinuous data.	[187]
		2D grid (64*64)	2D grid (64*64) for decoder Spectra (61) for encoder	24,000		MSE 0.001			[189]
	GAN	2D grid (64*64)	Transmission curve (128)	6500	10 m	Average absolute error 0.01	Generation is more powerful than AE and can generate multimodal samples. Stabilizes the training process, allowing multiple inputs to be processed in parallel, allowing for a more comprehensive treatment of the design problem.	More complex training processes and higher demands on training time and resources. And the effect of training is difficult to assess directly. Defining the Wasserstein distance is difficult and requires imposing complex mathematical constraints.	[192]
		EM response + noise (100 + N)	2D grid (64*64)	29,000	72 h				[193]

Numbers in parentheses indicate the dimension of data. GP represents geometry parameters. For runtime, s indicates seconds, m indicates minutes, h indicates hours, and d indicates days.

## 4 AI for metasurface systems

Metasurface, known for their advantages such as lightweight, compactness, and easy integration, have been widely used in various optical systems to achieve numerous customized functionalities, including optical imaging, holographic display, structured light generation, and polarization control. As the research framework of metasurface gradually matures, researchers have put forward higher requirements for various systems based on metasurface. The introduction of AI enables these systems to be more precise, adaptive, and multifunctional to meet the needs of different fields, leading to significant advancements in imaging, sensing, and novel systems. In this section, we primarily introduce several common metasurface systems and their developments when combined with AI.

### 4.1 AI-enhanced imaging systems

Metasurface imaging systems are complex systems composed of metasurface and other optical components. They can capture and process the optical information of target objects or scenes to generate images. Based on the different functionalities of metasurface, traditional imaging systems can be classified into several categories: diffractive imaging utilizes the metasurface’s ability to control arbitrary wavefront transformations for high-resolution image acquisition [200], [201], [202]; polarization imaging achieves more efficient and compact polarization image acquisition through the polarization-dependent characteristics of nanoscale antennas in metasurface [203], [204], [205]; and spectral imaging modulates the incident electromagnetic waves using metasurface to disperse and collect light of different wavelengths, thus obtaining richer information.

Computational imaging, as a novel imaging technique, emphasizes the use of computers to enhance and optimize the process of image acquisition, combined with physical processes, thereby improving image quality, resolution, and information extraction efficiency of imaging systems. In 2023, Shen et al. [206] designed and demonstrated a compact monocular camera equipped with a single-layer metalens. This metalens design incorporated a pair of polarization-decoupled rotating single-helix point spread functions. By combining a simple physically informed image reconstruction algorithm, the camera achieved high-precision depth measurement and high-fidelity polarization imaging in dynamic indoor and outdoor scenes. With further advancements in computer technology, artificial intelligence techniques such as machine learning and compressed sensing have become one of the most effective means to enhance metasurface imaging.

#### 4.1.1 High quality imaging

Realizing high-quality imaging using metasurface is a key technology aimed at obtaining clearer, more accurate, and detailed images, and it has been widely applied in fields such as science, medicine, engineering, and others. AI plays an important role in high-quality imaging by leveraging its powerful image processing and analysis capabilities to assist in achieving higher resolution, contrast, and accuracy.

**Figure 9:**
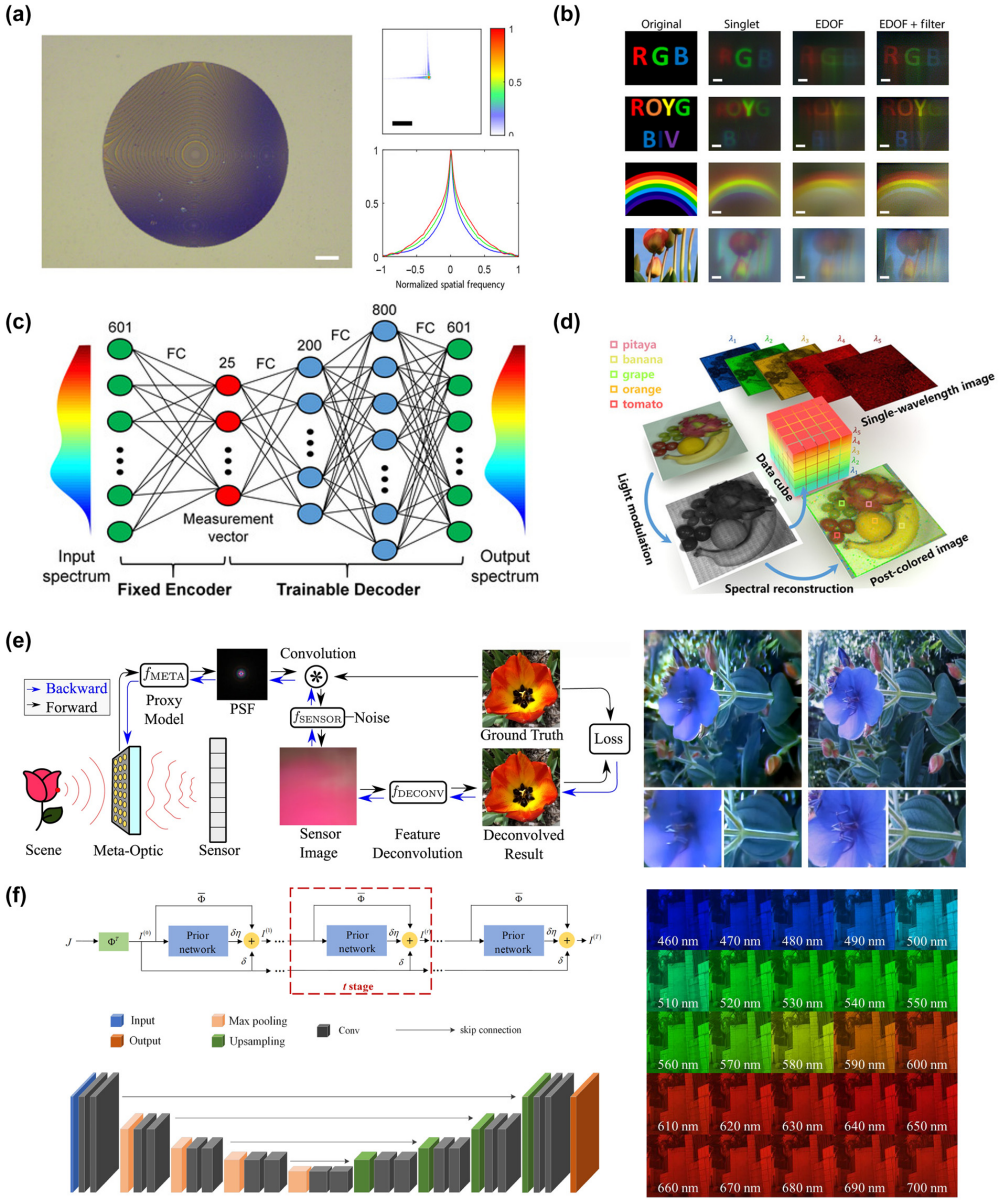
AI-assisted high-quality imaging. (a) EDOF metalens can extend sufficient depth of focus using a cubic phase mask. On the sensor, we can see that the green light is defocused in this plane. The MTFs of EDOF metalens are similar for all wavelengths and have higher cutoff spatial frequencies than red and blue metalens [207]. (b) Landscape image of text and colorful pattern under white light illumination captured by EDOF metalens and reconstructed by Wiener filter [207]. (c) Schematic diagram of the spectral encoder–decoder network [34]. (d) A 356 × 436 × 601 data cube of a plate of fruit spectral images is reconstructed from the metasurface modulated original image [34]. (e) The entire end-to-end imaging process is composed of a metasurface imaging process model and a neural deconvolution algorithm. The image on the right shows a blue flower reconstructed by the proposed neural nano-optics [41]. (f) The combination of deep neural network and hyperspectral image prior network forms the overall architecture of hyperspectral image reconstruction. With the help of this network, the experiment reconstructed hyperspectral images of 25 spectral channels from 460 nm to 700 nm. Reproduced from [208]. Copyright 2023, Elsevier.

In common back-end image processing, machine learning learns and captures patterns and features of images from a large amount of training data, enabling tasks such as denoising, image restoration, autofocus, object detection, and image classification. Many machine learning techniques have achieved remarkable results in spectral reconstruction processing of metasurface imaging systems. In 2018, Colburn et al. [207] designed a metalens with extended depth of focus (EDOF) that produced spectrally invariant blur, with increasing bandwidth as the depth of focus increased, showed in Figure 9(a). To obtain accurate spectral information from the blurred images, the authors performed preprocessing on the captured images and then utilized a computational deconvolution method for reconstruction, achieving high-quality wideband full-color imaging under white light illumination, as shown in Figure 9(b). This approach, which optimizes metasurface chromatic aberration through algorithms, had provided new insights for the design of various optical systems. Yang et al. [34] employed freeform-shaped meta-atoms for on-chip hyperspectral imaging. As shown in Figure 9(c) and (d), to evaluate the spectral reconstruction performance of the selected transmission spectra at different minimum feature sizes, the authors constructed a spectral encoder–decoder network. They used synthesized Gaussian linear spectra as input spectral datasets and simulated the spectral measurement process using the encoder, followed by training the spectral decoder for spectral reconstruction. In this work, the imaging system achieved a spectral resolution of 0.5 nm, with an average fidelity of 98.78 % for spectral reconstruction on a standard color chart. The spatial resolution was 356 × 436, demonstrating excellent performance.

In addition to direct back-end processing of imaging, the entire imaging process can be viewed as a unified entity, known as “end-to-end” design. By introducing the design parameters of metasurface and the output image parameters as variables to be optimized, the imaging system can be connected from the front-end to the back-end, achieving both structural and image optimization through a complete AI workflow [34]. Tseng et al. [41] proposed neural nano-optics and designed and validated a high-quality, polarization-independent metasurface imaging system using an end-to-end architecture. The imaging process was divided into three parts: metasurface phase determination, PSF simulation and convolution, and sensor noise. This imaging system achieves simulation accuracy comparable to FDTD but with a speed advantage of three orders of magnitude and a memory requirement reduced by 3000 times. Unlike conventional methods, the neural deconvolution approach was applied to the learned feature space rather than the original image intensity during the back-end reconstruction. This technique combined model-based deconvolution generalization with effective feature learning in neural networks, enabling image deconvolution of meta-optics with severe aberrations and large spatial range PSFs. As shown in Figure 9(e), the simulation of the imaging process and the deconvolution algorithm constituted a fully differentiable end-to-end design chain, resulting in a faster and higher-quality metasurface imaging system. After training and optimization, this nanophotonic imaging device reconstruction method generated RGB images of 720 px*720 px in just 58 ms, with a MSE 10 times lower than existing methods, and achieved a spatial resolution of 214 lp/mm in all color channels at a distance of 120 mm. Similar end-to-end algorithms have also been used to design a compact and portable instantaneous hyperspectral imaging system [208]. To achieve high-quality spectral reconstruction from RGB three channels to multiple channels, a spectral prior-unfolding neural network was introduced. The network for computing the spectral prior consisted of a classic U-Net [209], which learned how to extract spectral details from a large training dataset of hyperspectral images. Figure 9(f) shows the overall network. In the iterative training process, the input data passed through the prior network to generate a high-quality spectral image prediction, continuously optimizing the loss between the computed and real images. Based on this end-to-end algorithm, the experiments demonstrated better hyperspectral reconstruction results compared to traditional separate optimization methods, showing significant advantages in terms of average peak signal-to-noise ratio (PSNR). AI techniques automate and optimize each step, simplifying the process and improving efficiency, leading to remarkable advancements in metasurface imaging systems, potentially becoming the next generation of high-quality cameras.

#### 4.1.2 Multidimensional imaging

AI-assisted metasurface imaging systems have made significant achievements in providing high-quality imaging. However, to further enhance our understanding and analytical capabilities of complex problems, multidimensional imaging becomes particularly crucial. Multidimensional imaging not only captures more information but also provides different perspectives, revealing a more comprehensive image. AI-assisted multidimensional imaging can innovatively integrate multidimensional data into a unified framework and analyze, interpret, and extract useful information using deep learning and pattern recognition techniques, thereby achieving 3D and even 4D imaging.

**Figure 10:**
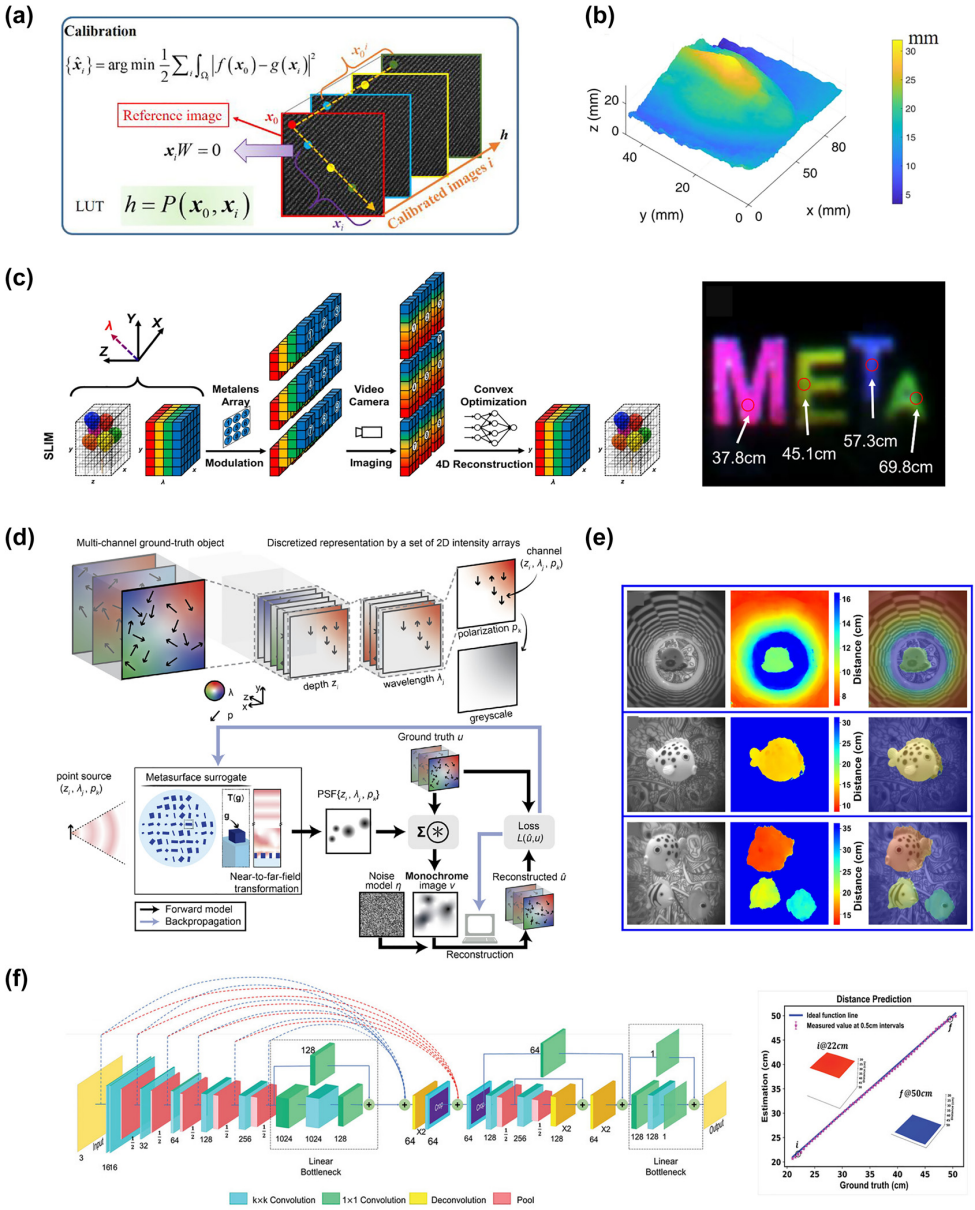
AI-assisted multidimensional imaging. (a) Calibration algorithm. A single illumination stripe can create spatial ambiguity due to the single-item coding properties. This algorithm calibrates sub-pixels through geometric constraints and similarity requirements of *x*_*i*_, thereby establishing the relationship between depth *P* and (*x*_0_, *x*_*i*_) [210]. (b) Perspective view of reconstructed facial 3D imaging [210]. (c) SLIM system. The 4D data cube of “*x* + *y* + *z* + *λ*” is decoupled into multi-view aliased information through modulation of the metalens array and captured by the camera. Finally, the spectral reconstruction algorithm and super-resolution module proposed in the article are used to achieve 4D imaging. On the right is a scene captured by SLIM consisting of four letters of different spatial positions and colors [42]. (d) Multichannel end-to-end design. Multichannel ground-truth images can be continuously cut: 3D objects are divided into sets of multiple 2D depth slices, 2D depth slices can be cut into sets of different color slices, and different color slices can be cut into sets of different polarization slices. Applying these slices to the end-to-end architecture results in an overall multichannel optimization. Reproduced from [200]. Copyright 2022, Optica Publishing Group. (e) Underwater depth-sensing results. The left column is the raw image captured by the left meta-lens. The middle column is the depth map. The right column is the integration of two images [211]. (f) The left picture is architecture of the neural networks for light-field net (LFN). The right picture is variation of distance measured by light-field versus the known reference of the stairs. Reproduced from [212]. Copyright 2023, Wiley Materials.

Jing et al. [210] designed and validated a system for active 3D positioning and imaging using single-frequency fringe illumination. The researchers introduced a metasurface as an encoded light source into the imaging system, utilizing geometric phase design to encode fringes and obtaining deformed fringe images in reflective scenes during experiments. As shown in Figure 10(a), to address the depth ambiguity of the single-fringe approach, the article introduced a calibration model with polar constraints and similarity search and employed the alternating direction method of multipliers (ADMM) algorithm to achieve depth reconstruction with depth deviations smaller than 0.5 mm within 300–400 mm. Furthermore, a correspondence between depth and image coordinates was established, ultimately achieving high-precision 3D imaging, showed in Figure 10(b). Hua et al. [42] realized an imaging system that captures both the light field and spectral information in a single shot using a lateral-dispersion metalens array. This ultra-compact spectral light-field imaging (SLIM) system consists of a 48 × 48 array of superchromatic metalens and a monochrome CMOS sensor, where different wavelengths of the spectral light field overlap on a 2D receiving plane, causing blurring in the captured patterns. To utilize the additional information from these blurs and obtain complete spectral information, the authors proposed an algorithm for super-resolution spectral reconstruction. Figure 10(c) shows the flowchart. This algorithm transformed the reconstruction task into a solved convex optimization problem, effectively recovering high-quality multispectral images from monochrome blurred images by combining a forward model established based on the design parameters of the metalens and regularization constraints. To achieve higher spectral resolution, the researchers trained a spectral super-resolution network using paired low-resolution and high-resolution spectral data as input and output, placing this network as a super-resolution module after the reconstruction algorithm. Based on this algorithm, SLIM improved the spectral resolution to 4 nm and obtained depth information by calculating the disparity between adjacent metalens, enabling “*x* + *y* + *z* + *λ*” 4D imaging. In the experiments, the authors further processed the spectral images, such as differential amplification to distinguish peaks, effectively differentiating objects with similar spectra but different materials based on depth information.

The aforementioned end-to-end design can also be assisted by improved algorithms for multidimensional imaging. Lin et al. [200] combined Tikhonov regularization with an end-to-end architecture for inverse design of metasurface and replaced the backend with compressive sensing, thereby increasing channel capacity at the cost of stronger priors. As shown in Figure 10(d), they optimized the design of a single nanophotonics structure and achieved reconstruction of multiple depth, spectral, and polarization channels from a single monochromatic image. In the experiments, the metasurface design of a 16-color imager achieved a reconstruction error of 5–12 % (lower than 1 % image noise), the 4-color/4-depth imager had an error of 5 %, and the 2-color/2-depth/4-polarization imager had an error of 2 %.

AI has also facilitated multidimensional imaging in special environments. In recent years, Tsai’s research team has developed a new underwater binocular stereo vision and deep sensing system [211], which incorporates AI-supported GaN binocular metalens. This system meets the imaging requirements for underwater applications, such as compactness, lightweight design, and low power consumption. As shown in Figure 10(e), they propose a generalized depth calculation formula applicable to binocular vision systems of various sizes. Combined with deep learning support, real-time processing capabilities for rapid underwater object imaging and depth calculation are achieved, with a depth measurement accuracy of up to 50 μm. Additionally, the superhydrophobic nature of the metalens endows the underwater binocular system with properties such as antiadhesion, pollution resistance, and self-cleaning, making it highly promising for wide applications in underwater robotics, submarines, and marine machine vision. Furthermore, their team has recently developed an intelligent meta-device [212] that integrates light field imaging and structured light systems, utilizing AI algorithms to achieve depth perception, particularly excelling in low-light environments. On one hand, in the light field–based depth perception mode, AI algorithms are used to process the light field images captured by the metalens array, generating depth maps, and conducting more in-depth image analysis through neural networks. On the other hand, in the chromatic aberration-based structured light system, the meta device adopts a structured light pattern, such as structured light patterns generated by a 532 nm laser. As illustrated in Figure 10(f), the article employs deep learning algorithms and introduces a structured light network (SLN) for semantic recognition of the structured light images. The entire system achieves precise perception of object depth under low-light conditions, demonstrating remarkable adaptability.

### 4.2 AI-enhanced sensor systems

Metasurface can be designed to be particularly sensitive to small variations in specific physical parameters, making them suitable for creating highly sensitive sensors (metasensors) [213]. Metasensor systems consist of metasensors, data acquisition, signal processing, and data output, and they are widely used in fields such as biology, chemistry, and optics to perceive and understand the properties and characteristics of substances and motion. AI plays a crucial role in metasensor systems, enabling rapid progress from sensor design to data processing.

#### 4.2.1 Material sensing

Metasurface technology plays a significant role in material sensing, particularly enabling high-precision spectral detection. This technology has been widely applied in the fields of biology and chemistry [214], [215], [216], [217]. In the field of biology, metasensors are commonly used to detect biomolecules, cells, microorganisms, and other biological entities. In the field of chemistry, metasensors are employed for analyzing chemical reactions and material properties. The precise spectral analysis provided by metasurface sensors holds great potential for important applications in medical diagnostics, environmental monitoring, and the study of new materials.

**Figure 11:**
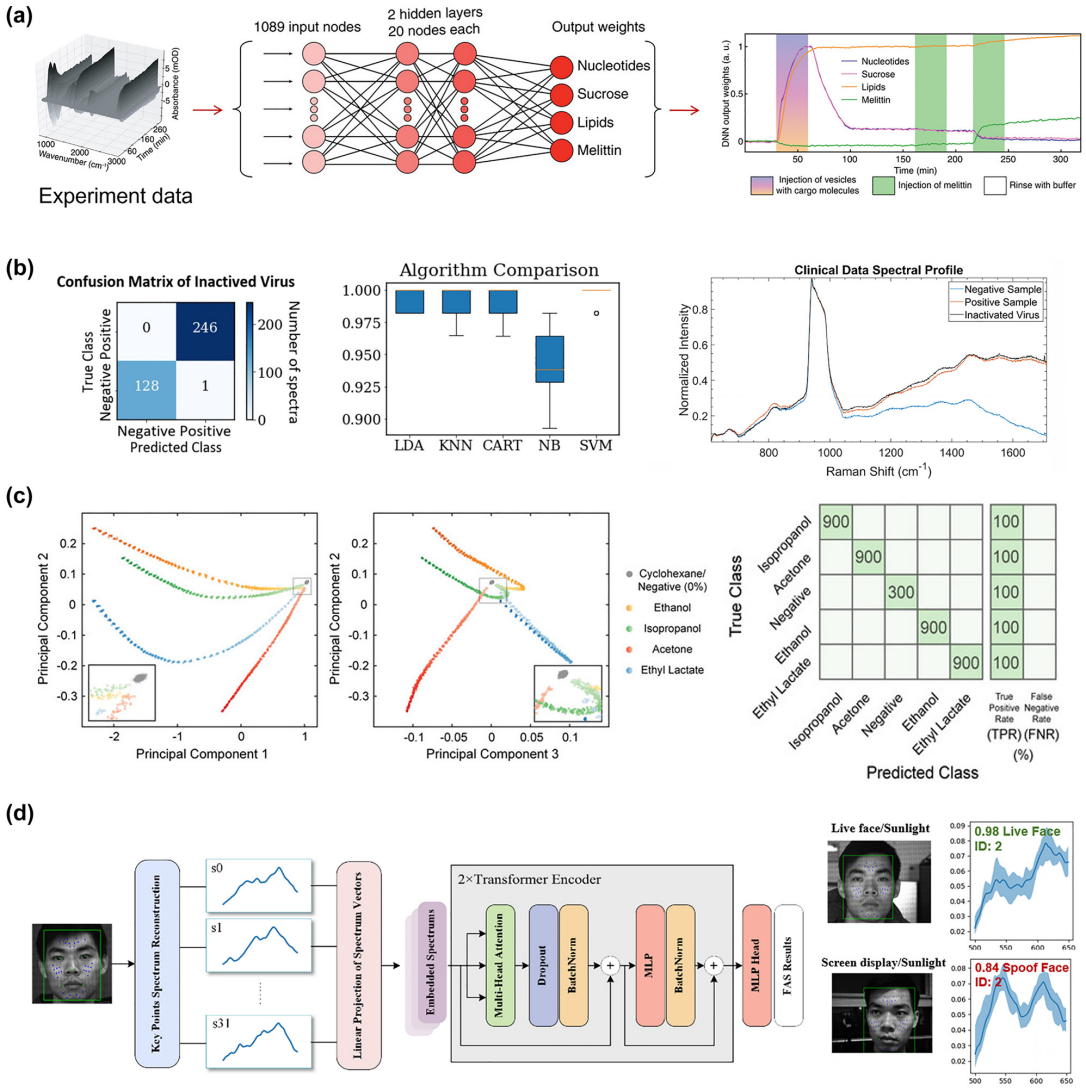
AI-enhanced material sensing. (a) Spectrotemporal data points from a dual cargo release experiment were fed into a real-trained DNN to predict the scaled DNN output weight curves for each analyte [218]. (b) In order to find the best model for virus classification, 5 different algorithms were tested using KFold cross-validation (*k* = 10). During training, the SVM model was used to measure the confusion matrix of the sample Raman spectrum. In clinical testing, this metasurface sensor successfully differentiated samples collected and compared their average spectra with inactivated virus samples [219]. (c) Through PCA of the data after interpolation, the experiment finally obtained retained data points to perform concentration verification and retain the verification results in the confusion matrix. Reproduced from [220]. Copyright 2022, Wiley Materials. (d) Architecture of the FAS classifier. By adopting the self-attention mechanism using transformer encoder, not only are the features of each spectrum sample analyzed respectively but also the cross-correlations of 32 samples are taken into account. Finally, the output vector by transformer encoder is sent to a MLP to get the final FAS results. The figure on the right shows the results of a live face captured and the face results displayed on the screen under sunlight, verifying its antispoofing recognition capabilities in the real world [221].

In the field of biology, mid-infrared spectroscopy is often utilized for identifying various biomolecules such as proteins, lipids, nucleic acids, and polysaccharides. To meet this demand, researchers, such as John-Herpin et al. [218], have designed a broadband nanoplasmonic metasurface for surface-enhanced infrared absorption spectroscopy (SEIRAS). By tuning multiple resonant peaks, they covered the absorption range from below 1000 cm^−1^ to above 3000 cm^−1^. As shown in Figure 11(a), they constructed a fully connected neural network model with an input layer of 1089 nodes and an output layer of 4 nodes. The training set consisted of over 3 million spectral data points obtained from various concentrations of four analytes. The trained deep neural network model, integrated with a microfluidic chip-based metasensor, formed a sensing system capable of real-time monitoring of lipid vesicle capture, cargo release induced by melittin (a small cell lytic peptide), and the transition of partial lipid vesicles to support lipid bilayer (SLB). In addition to mid-infrared spectroscopy, Raman scattering spectroscopy can also be employed for analyzing molecular structures and chemical compositions. Ahmadivand et al. [222] utilized GA to design and optimize metasurfaces for surface-enhanced Raman scattering (SERS) substrates, enabling the detection of SARS-CoV-2 virus. To analyze the SERS data, the authors collected hundreds of Raman spectra from different samples. After preprocessing steps such as normalization, feature scaling, and Savitzky–Golay filtering, the data dimension was reduced using PCA algorithm. In the experiments, a supervised learning model, SVM, was trained using data from 15 PCR-positive samples and 12 PCR-negative samples for virus identification. The results demonstrated that this metasurface sensor combined with a machine learning model could accurately detect the presence of the virus in clinical samples, even in the presence of highly heterogeneous backgrounds, such as saliva samples, shown in Figure 11(b).

In the field of chemistry, the identification capability of mid-infrared spectroscopy has been applied in industrial production, food production, and environmental monitoring of chemical substances. Meng et al. [220] developed a compact mid-infrared spectrometer for the identification and quantification of chemical substances. The plasma metasurface, composed of gold nanostructures on an undoped silicon substrate, was integrated into a filter chip containing 10 bandpass filters and 10 bandstop filters, covering the mid-infrared range of 6–14 μm. The entire microspectrometer system consisted of the filter chip and a microbolometer thermal imaging sensor. As shown in Figure 11(c), by collecting the sensor outputs of different concentrations of chemical solutions and using machine learning algorithms such as PCA and SVM for training, a 100 % classification accuracy was achieved for ethanol, isopropanol, acetone, and ethyl acetate in cyclohexane. Furthermore, this mid-infrared microspectrometer combined with machine learning algorithms enabled the effective identification and quantification of various complex samples, such as three drugs (acetaminophen, ibuprofen, and aspirin) with a 100 % discrimination accuracy and three edible oils (olive oil, rapeseed oil, and peanut oil) with a discrimination accuracy ranging from 94 % to 100 %.

The application of AI in material sensing systems provides examples of data analysis and model training, which are also crucial for novel biometric technologies such as facial recognition. Rao et al. [221] achieved facial recognition through a snapshot hyperspectral imaging sensor based on metasurface nanostructures. This imaging sensor accurately measured the facial reflectance spectra and revealed the absorption peaks of hemoglobin. In the experiments, this sensor, combined with convex optimization methods and compressed sensing algorithms, constructed a practical antispoofing facial system, showed in Figure 11(d), achieving an accuracy of 97.98 % in real-world testing scenarios.

AI accelerates data processing, improves recognition accuracy, and identifies subtle signals and patterns, making material sensing systems more intelligent and adaptive.

#### 4.2.2 Motion sensing

By manipulating the electromagnetic (EM) wave field, metasurface can detect dynamic parameters such as object position, velocity, and direction. The application of AI enables motion sensing systems to process a large amount of dynamic data in real-time, recognize complex motion patterns and trends [223], [224], and thus, it has important value in applications such as intelligent surveillance, robot navigation, gesture recognition, and virtual reality (VR).

**Figure 12:**
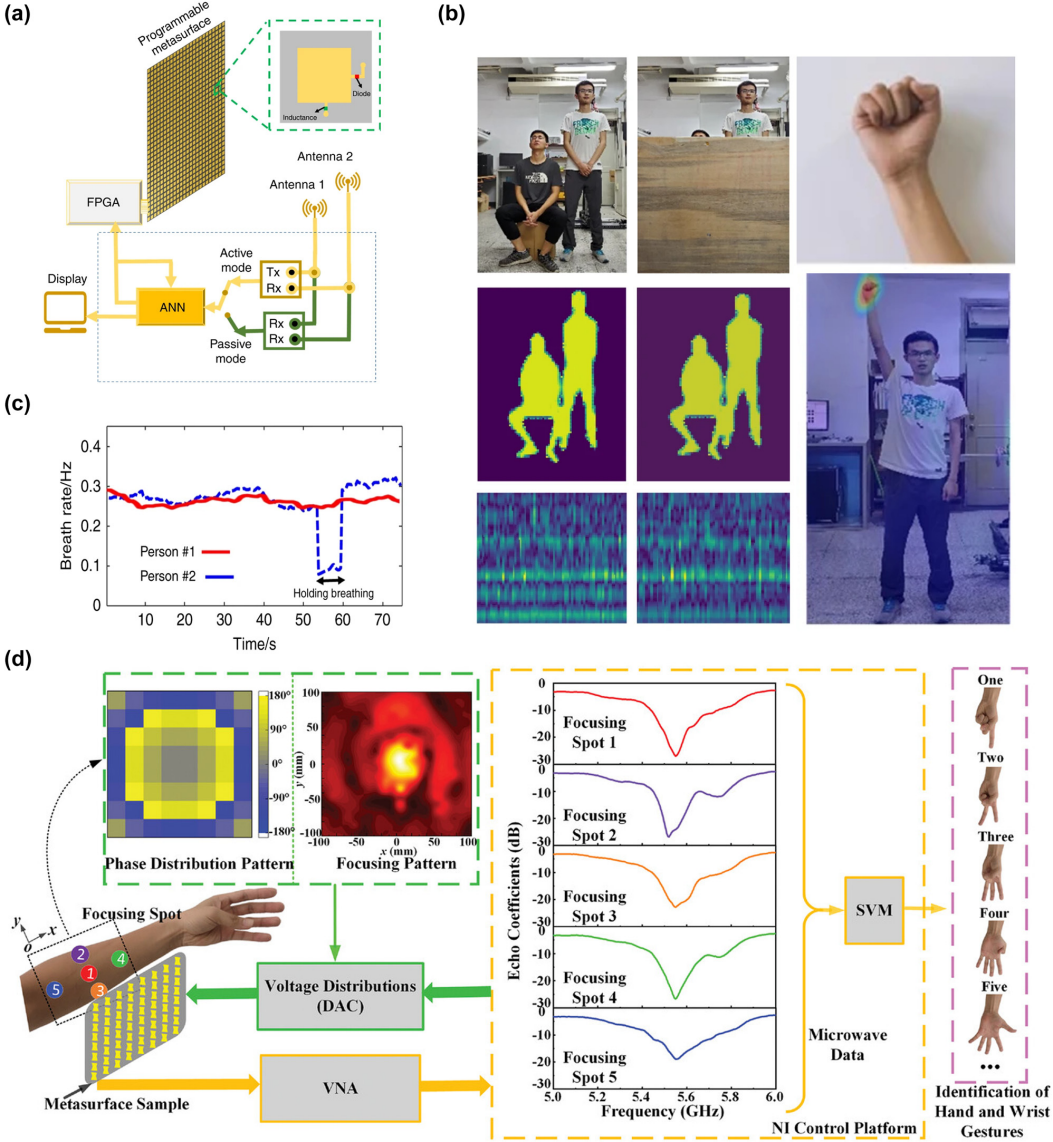
AI-enhanced motion sensing. (a) The schematic configuration of intelligent metasurface system that uses a large-aperture programmable metasurface to adaptively manipulate and sample electromagnetic wave fields and to instantaneously control and process data streams [202]. (b) IM-CNN-2 processes data to recognize gestures and uses active microwave metasurface combined with IM-CNN-1 to achieve imaging behind a wooden wall [202]. (c) Identification of human respiration through time-frequency analysis of microwave data [202]. (d) The working principle of the noncontact hand gesture recognition method based on the programmable metasurface sample with a machine learning algorithm. The five different voltage distributions corresponding with phase distribution patterns can be obtained for controlling the transmissive programmable metasurface so that its radiation wave is focused onto the desired spots [225].

In 2019, Cui’s research team [202] integrated programmable metasurfaces with deep learning techniques to create an intelligent metasurface recognizer. As shown in Figure 12(a), this programmable metasurface consisted of 24 × 24 digital meta-atoms, with each meta-atom integrating a PIN diode for control. The entire recognition system consisted of three ANNs, where IM-CNN-1 was a CNN that transformed the received electromagnetic wave data into an image encompassing the entire human body, providing a data foundation for subsequent processing. FASTER R-CNN was a commonly used object detector that identified the positions of hands and chests in the entire image, enabling more accurate tracking of gestures and breathing actions. IM-CNN-2 was another CNN used to directly process the raw electromagnetic wave data. Experimental results are showed in Figure 11(b) and (c), which demonstrated that this metasurface sensing system achieved various precise recognition and detection functionalities: accurate recognition of different hand poses such as stretching and fist clenching, differentiation of normal breathing and breath-holding states, and even detection of movements behind obstacles 5 cm thick by analyzing the reflections of microwave signals. There is also an intelligent prosthetic recognition system based on noncontact electromagnetic sensing [225]. Programmable metasurface technology was employed to perceive limb movements by manipulating the focus of electromagnetic waves. Researchers focused electromagnetic waves on the measured arm to obtain echo data associated with different gestures and actions. After cleaning and standardizing the data, feature extraction was performed using Fisher scores to ensure its quality and practicality. As shown in Figure 12(d), the recognition system trained the collected dataset using the One-vs-One Support Vector Machine (OVO-SVM) algorithm to differentiate different gestures and actions. Experimental results demonstrated successful classification and recognition of 18 hand gestures, including hands and wrists, by the programmable metasurface after training. It also showed that using multiple foci improved both classification accuracy and sample quantity compared to a single focus. These studies open up more possibilities for AI-integrated metasurface sensing systems in areas such as smart homes, medical detection, and security screening.

### 4.3 AI-enhanced holographic systems

As a technology for recording and reproducing three-dimensional information of light waves, holography achieves comprehensive recording of real scenes by capturing the phase and amplitude information of light waves. With the advancement of computers, traditional holography has evolved into digital holography, which converts optical information into digital information, thereby enhancing the flexibility and efficiency of image processing.

The integration of metasurfaces and digital holography in metasurface holographic systems has brought about innovative advancements. By encoding computer-generated holograms (CGH) onto metasurfaces, researchers have opened up new possibilities for personalized holographic rendering [106], [226], [227], [228], [229]. Building upon this innovation, the introduction of AI further enhances digital holography. In recent years, AI has proven to be useful in improving holographic imaging techniques, such as mapping color and depth images to corresponding holograms using convolutional neural networks [230]. By leveraging the advantages of AI in metasurface design and digital holographic computation, metasurface holographic systems not only adapt better to different scenarios and requirements but also achieve higher levels of optical performance and image reconstruction quality.

**Figure 13:**
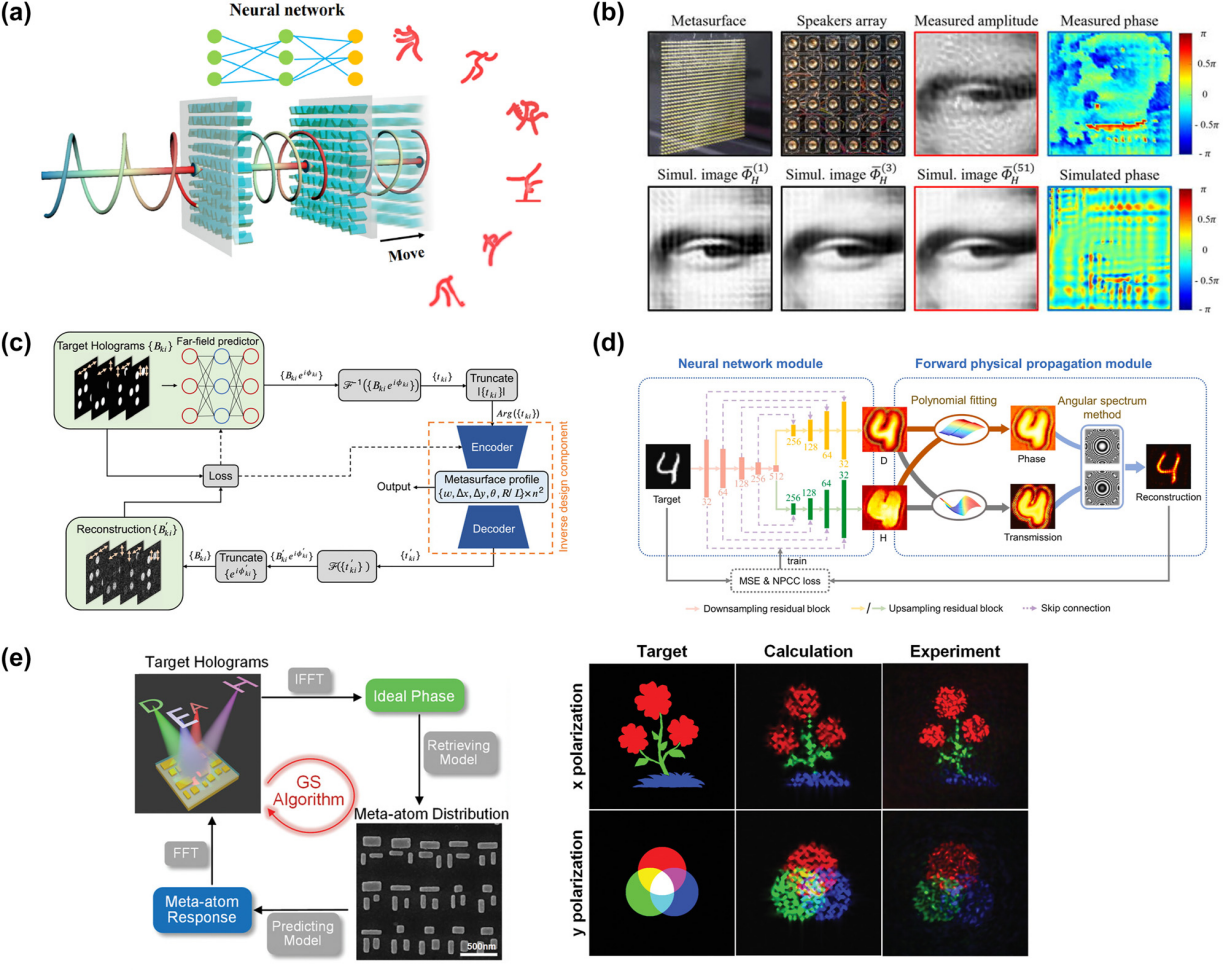
AI-enhanced holographic system. (a) Schematic diagram of ultra-dense cascaded metasurface holography. The two layers of metasurface can be regarded as a diffraction-like neural network, and the complex amplitudes of the two layers are obtained through reverse design [231]. (b) Measurement of the eye part of the Mona Lisa hologram, using a 6 × 6 speaker array as the sound source, and passing through a metasurface sample of 30 × 30 3D printed components to measure the amplitude distribution and phase distribution. The second row corresponds to the calculated holographic images of the 1st, 3rd, and 51st iterations from left to right. The last picture is the simulated phase distribution on the target image plane. Reproduced from [232]. Copyright 2023, AIP Publishing. (c) Integrated deep neural network to design metasurface hologram [233]. (d) Architecture of the network with cooperation of two modules, i.e., neural network module and forward physical propagation module. The output image of forward physical propagation module is reconstructed to the same pattern as the target image to obtain the diameter map and the height map of nanocylinder array [234]. (e) End-to-end iterative design flowchart. Embedding a statistical learning model into a GS algorithm for multifunctional metasurface holograms enables targeted, computational, and experimentally measured multicolor hologram images for *x*- and *y*-polarizations. Reproduced with [100]. Copyright 2022, Wiley Materials.

AI can enhance both metasurface design and hologram reconstruction individually, thereby strengthening the front-end and back-end of metasurface holographic systems. Zhou et al. [231] proposed and demonstrated an optical holographic encryption strategy using a mobile cascaded complex-amplitude metasurface system, significantly increasing the holographic encryption information capacity and storage density while ensuring image quality. As shown in Figure 13(a), the researchers constructed the cascaded system as a physics-based neural network, employing the Adam optimizer with a learning rate of 0.0001. They built the loss function by comparing the reconstructed holographic images with ground truth and iterated a finite number of times to obtain the complex-amplitude distributions of the two metasurfaces and the distance between them. The metasurface design was then based on these distributions. It’s worth noting that this cascaded system places significant alignment requirements on the metasurface, making experimental validation challenging. Miao et al. [232] proposed and validated an acoustic metasurface holographic system designed using deep learning and genetic algorithms. In their study, a fully connected neural network was employed to predict the phase and amplitude of the elements, and GA were used for iterative optimization, enabling highly flexible metasurface designs. More importantly, the authors presented an iterative compensation scheme to eliminate interference fringes and effectively improve the quality of holograms. As shown in Figure 13(b), this approach not only improved the efficiency and accuracy of metasurface holographic system designs but also showcased the possibility of frequency-selective imaging in practical applications. This method can also be extended to other wave-based meta-devices, including wavefront engineering and polarization control.

End-to-end algorithms can also be employed for holistic optimization of metasurface holographic systems. Xi et al. [233] introduced a deep learning algorithm called DeepCGH-ID for efficient hologram generation and enhanced the design process of optical metasurface holographic systems. As shown in Figure 13(c), through an encoder–decoder structure, this algorithm converts the given phase information of holograms into relevant nanoscale structure parameters, enabling automatic hologram design. This method exhibits significant advantages over traditional approaches in terms of generation speed and performance, especially when dealing with a large number of designs. Not only can this algorithm generate high-quality holograms, but it also possesses the capability for reverse design, providing an automated solution for complex design tasks. Similarly, Wei et al. [234] proposed an end-to-end network structure called Y-Net, which combines the generative module and physical propagation module of neural networks, enabling the design of optical metasurface holograms at subwavelength scales. As shown in Figure 13(d), this algorithm utilizes a Y-shaped network structure that includes downsampling and upsampling residual blocks, trained using loss functions such as mean squared error and negative Pearson correlation coefficient. In the physical propagation module, polynomial fitting and angular spectrum methods are employed to reconstruct the target electric field pattern. The neural network stabilizes convergence within 2 h, and once the training process is completed, metasurfaces can be designed in less than 1s. The article achieves end-to-end design from geometric parameters directly to high-quality holograms, providing robust support for real-time and large-scale holographic displays. Ma et al. [100] embedded statistical machine learning models into gradient or nongradient methods to achieve end-to-end system design for multifunctional metasurfaces. The researchers quantitatively described the interdependencies of optical responses at different frequencies using mutual information, thereby utilizing the statistical characteristics of metasurface structural units to drive the limits of design capacity. As shown in Figure 13(e), the authors integrated statistical machine learning models into the Gerchberg–Saxton algorithm for holograms, successfully generating eight metasurface holograms under different combinations of two orthogonal linear polarizations and four frequencies (270, 350, 375, and 400 THz) of illumination conditions. Additionally, the authors used this model to design polarization multiplexed multicolor holograms, enabling the projection of different colored patterns under two different polarizations. This end-to-end framework, incorporating statistical machine learning, opens up a new path for efficient and multi-objective design of metasurface holographic systems.

### 4.4 AI-enhanced novel systems

Through intelligent analysis, adaptability, and real-time control, AI can empower various innovative metasurface technologies with new capabilities. Next, we will explore how AI can enhance the functionality of various novel metasurface systems to meet the needs and challenges in different fields.

**Figure 14:**
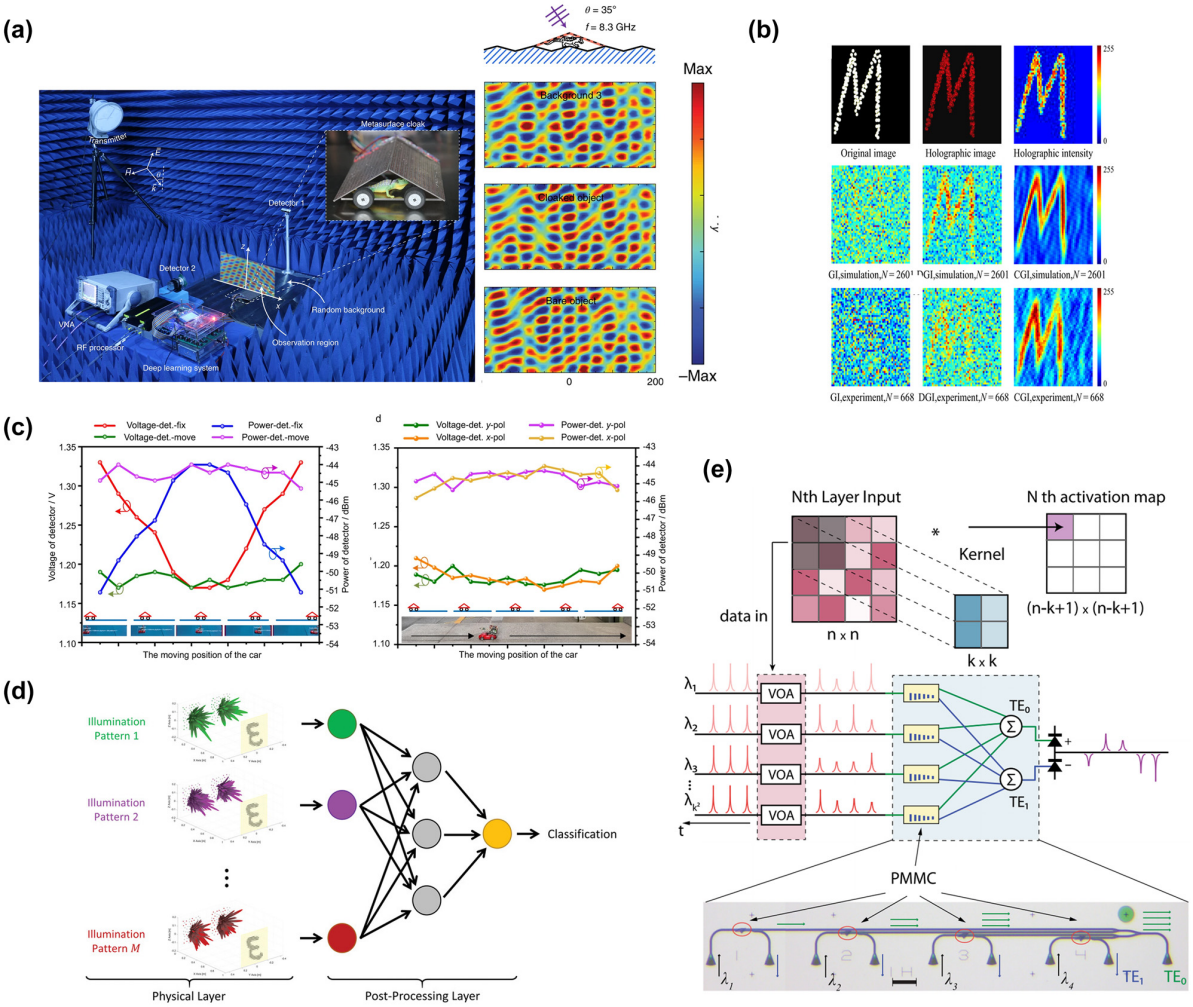
AI-enhanced novel systems. (a) Experimental setup. A TM-polarized Gaussian beam from a high-directivity lens antenna is incident on the scatterer with incident angle *θ*. The two detectors detect the surrounding background and incident waves in real time, input them into the deep learning system, calculate the results in time, and provide them to metasurface cloak. Reproduced from [235]. Copyright 2020, Springer Nature. (b) Simulated and experimental results with different GI algorithms. This confirms the great advantages of CGI in imaging reconstruction [201]. (c) Indoor and outdoor radio frequency signal detection experiments. The first picture shows the radio frequency signal changes when the detector is fixed in the middle of the path or moves with the car. The second picture shows the received radio frequency signals under *y*-polarization and *x*-polarization when the detector moves with the car [240]. (d) Sensing protocol. The scene is illuminated with M distinct TX-RX metasurface configurations, yielding a 1 × M complex-valued measurement vector that is processed by an artificial neural network consisting of fully connected layers. The output is a classification of the scene [244]. (e) Using a PMMC array as a photonic computing core for convolutional image processing. The PMMC is programmed to store the kernel matrix and encode the input image into a light pulse input into the channel with the kernel [245].

Realizing arbitrary invisibility of objects has always been a desired function. An ideal invisibility cloak should be able to rapidly and automatically adjust its internal structure to adapt to different scenes, thus maintaining invisibility at all times. To pursue this extraordinary transformation, a reconfigurable metasurface cloak based on controllable diodes has been proposed [235]. The factors influencing the cloak (e.g., surrounding background, invisibility location) are converted into local reflection spectra and incident waves that generate nonlocal effects, which serve as inputs. The DC bias voltage used to control the reflection spectra of meta-atoms is considered as the output. Through training an ANN with 10,000 test samples, the relationship between incident waves, reflection spectra, and bias voltage is established. As shown in Figure 14(a), this metasurface cloak can rapidly respond to continuously changing incident waves and surrounding backgrounds within a millisecond time frame, demonstrating powerful adaptability.

“Correlated imaging,” also known as “ghost imaging,” is a special imaging technique [236]. It differs from traditional imaging methods as it does not require direct measurement of scattered photons but instead utilizes another set of photons to acquire information. However, its resolution is often limited by photon counting and poses challenges in data storage and processing. AI, as an optimal tool for accelerating the imaging process and improving imaging quality, naturally plays a crucial role. Liu et al. [201] achieved single-pixel ghost imaging of helicity-dependent metasurface holograms. Compressive sensing algorithms, combined with computational ghost imaging (CGI), were introduced based on the sparsity of the signal. In experiments showed in Figure 14(b), this technique provided advantages such as high resolution, wide-angle imaging, and simplified imaging setup. Additionally, researchers have employed the CGI algorithm in combination with metasurface ghost imaging systems to design an optical encryption scheme.

With the rapid growth of wireless data traffic, researchers have been delving into the possibilities of metasurfaces in wireless communication systems [237], [238], [239]. Among them, AI-assisted programmable metasurface have stood out for their adaptability and efficiency. Li et al. [240] designed a system for target tracking and wireless communication using a digital programmable metasurface (DPM). It leveraged a CNN for automatic detection of moving target positions in computer vision and a pretrained ANN integrated with polarization-dependent DPM for intelligent beam tracking and wireless communication. In experiments, the system achieved target tracking of a moving model car, detection of indoor/outdoor moving radio frequency signals, and high-speed information transmission of moving targets (Figure 14(c)). This concept of intelligent tracking with metasurfaces is paving the way for challenges in 6G wireless communication and the Internet of Things.

While AI enhances metasurface systems, it also brings new opportunities for AI itself. As computer technology continues to advance, the capacity for electronic hardware to transmit and process information is approaching its limits. Optical devices, utilizing photons as carriers for information transfer, have demonstrated the ability for ultrafast parallel processing in complex and lengthy tasks, making them one of today’s hottest research directions. ML algorithms, used to accelerate metasurface design, can also enable these trained metasurface systems to replace machine learning algorithms and form all-optical machine learning [241], [242], [243]. Hougne et al. [244] proposed “learning integrated sensing pipeline (LISP),” which simultaneously optimizes the physical layer (reconfigurable metasurface) and the neural network processing layer for microwave sensing tasks, showed in Figure 14(d). The physical layer is integrated into the artificial neural network pipeline and generates programmable microwave illumination patterns. In digital recognition experiments, both metasurface patterns and neural networks are jointly trained to maximize classification accuracy, resulting in a 10%–15 % improvement in accuracy with fewer measurement iterations compared to standard illumination strategies. This integration of programmable metasurface into the sensing layer greatly enhances the computational efficiency of machine learning. Apart from the sensing layer, metasurface can also participate in the training layer of machine learning, further driving the emergence of all-optical computing. Li’s research team [245] achieved a small-scale photonic convolutional neural network using a reconfigurable metasurface based on a phase-change material called GST. As shown in Figure 14(e), by programmatically modulating the structure of GST, precise control of the mode conversion between waveguide ET0 and ET1, which represents the weight parameters of the neural network, is achieved. The authors constructed a photonic core consisting of four such mode converters and realized image convolution. Experimental results demonstrated accurate matrix-vector multiplication calculations, as well as image edge detection and handwritten digit recognition. This demonstrates the potential of metasurface in enabling all-optical ML tasks.

In summary, AI can significantly enhance various metasurface technologies by providing intelligent control, optimization, and real-time adaptation. It can improve imaging quality and resolution in correlated imaging systems, enable adaptive beam tracking and communication in wireless communication systems, and facilitate the development of reconfigurable metasurface cloaks for object invisibility. Furthermore, AI can also be integrated into metasurfaces themselves, enabling all-optical machine learning and accelerating computational tasks. These advancements open up new opportunities for metasurfaces in diverse fields, including imaging, communication, sensing, and computing.

## 5 Conclusions and outlooks

Metasurface and AI, as the cutting-edge research directions in the fields of optics and computer science in the 21st century, have demonstrated a strong trend of interdisciplinary integration. This review provides a brief introduction to the basic concepts of metasurface and AI, with a particular focus on how AI has propelled the development of metasurface.

The unit design of metasurface has always been a challenging problem due to the high degree of freedom in its surface structure. To fully unleash the potential of metasurface, it is necessary to alleviate the computational burden. The introduction of AI has enabled us to explore more diverse structural designs for specific properties. From classical machine learning algorithms to the emerging dominance of deep learning algorithms, from supervised learning to unsupervised learning, suitable AI algorithms can reduce the conventional electromagnetic field simulation and optimization time, thereby enhancing research and development efficiency. One of the major challenges faced by AI-enhanced metasurface is the size of the dataset. As the design space’s DoF increase, the potential exponential growth of data and the highly nonlinear relationship between inputs and outputs pose challenges to the generalization capability of learning algorithms. Currently, fitting low-dimensional inputs and outputs yields good results, but the capability of simple algorithms in high-dimensional scenarios still needs further exploration. A general solution is to expand the sample size, but it may still be limited by the time required to generate a sufficient number of samples. In addition, Reinforcement Learning (RL) [246], [247] has the potential to address this problem. As a third approach beyond supervised and unsupervised learning, RL emphasizes the interaction between the agent and the environment to improve the agent’s state.

It can be observed that in many cases, algorithms are not interoperable, and there is no universal learning algorithm that applies to all types of research. Researchers may spend a considerable amount of time searching for suitable algorithms. Selecting and combining algorithms while ensuring effective capacity is challenging. Moreover, algorithms themselves require the tuning of a large number of parameters, and determining the quantity and range of these parameters is a problem that cannot be directly described by mathematical theory. It requires researchers to rely more on their “experience” to debug them. Transfer learning (TL) [248] is one possible direction that could address this problem. It leverages the similarity between data, tasks, or models to transfer previously learned knowledge to new scenarios. Additionally, Multi-Modal Machine Learning (MMML) [249], [250] can integrate various types of information, such as images, videos, and text, to process multimodal information from multiple sources. They are all crucial steps toward true AI and are expected to further enhance the efficiency of metasurface design.

AI has not only accelerated the unit design of metasurface but also driven and expanded various applications and developments in optical systems based on metasurface. In imaging systems, AI enables high-quality image reconstruction, multidimensional imaging, and end-to-end optimization, resulting in higher resolution and contrast. In sensing systems, AI achieves precise and intelligent spectral detection in material sensing and dynamic detection and recognition of parameters in motion sensing. In other novel systems, AI enhances the functionality of systems such as stealth cloaks, correlation imaging, wireless communication, enabling adaptive control, and greatly expanding the application scenarios of metasurface. Excitingly, metasurface also provide a new hardware platform for AI in the field of hardware and open up new directions such as all-optical computing.

In recent years, the development of digital information has also provided new directions for the advancement of metasurface. The concepts of digital coding and programmable metasurface have matured in recent years. Programmable metasurface can be efficiently manipulated for different tasks through artificial control. When AI collides with programmable metasurface, it brings higher adaptability and autonomy to metasurface systems. Exploring a generalized AI system architecture will be one of the directions to promote the development of programmable metasurface. In most metasurface optical systems, AI is typically used for backend processing, which leads to a disconnect between system functionality and the metasurface itself, thereby affecting the overall performance of the system. To address this disconnect, it is worth further exploring end-to-end design and optimization processes using deep learning. By optimizing the parameters of the entire system comprehensively, it is possible to achieve comprehensive intelligence and automation of metasurface from structural design to solving practical problems.

Metasurface-based systems are currently not widespread, and their main challenges lie in limited low-cost batch manufacturing capabilities, the influence of complex real-world conditions, and integration processes with other components such as chips. Overcoming these factors is necessary to make metasurface truly the leaders of the next generation of optical devices. Furthermore, strengthening the cross-disciplinary fusion of different fields such as optics, materials, biology, chemistry, and electronics will also drive continuous innovation and progress in AI and metasurface technologies in new demand spaces.
